# Supplementary material for: Deep single-cell decoding of human pancreatic islets reveals T2D β-cell gene expression defects
Source: EMBO J. 2026 Apr 15;45(11):3978–4005. doi: 10.1038/s44318-026-00744-w (PMC13226668; doi:10.1038/s44318-026-00744-w)
Supplement: Supplementary file 2 — Appendix [file 44318_2026_744_MOESM2_ESM.pdf]

Appendix

Deep single-cell decoding of human pancreatic islets reveals T2D β-cell gene expression defects

Table of content

**Appendix Figures S1-S16**	**Page numbers**
Appendix Figure S1	1
Appendix Figure S2	2
Appendix Figure S3	3,4,5
Appendix Figure S4	6
Appendix Figure S5	7
Appendix Figure S6	8
Appendix Figure S7	9
Appendix Figure S8	10
Appendix Figure S9	11
Appendix Figure S10	12
Appendix Figure S11	13
Appendix Figure S12	14
Appendix Figure S13	15
Appendix Figure S14	16
Appendix Figure S15	17
Appendix Figure S16	18

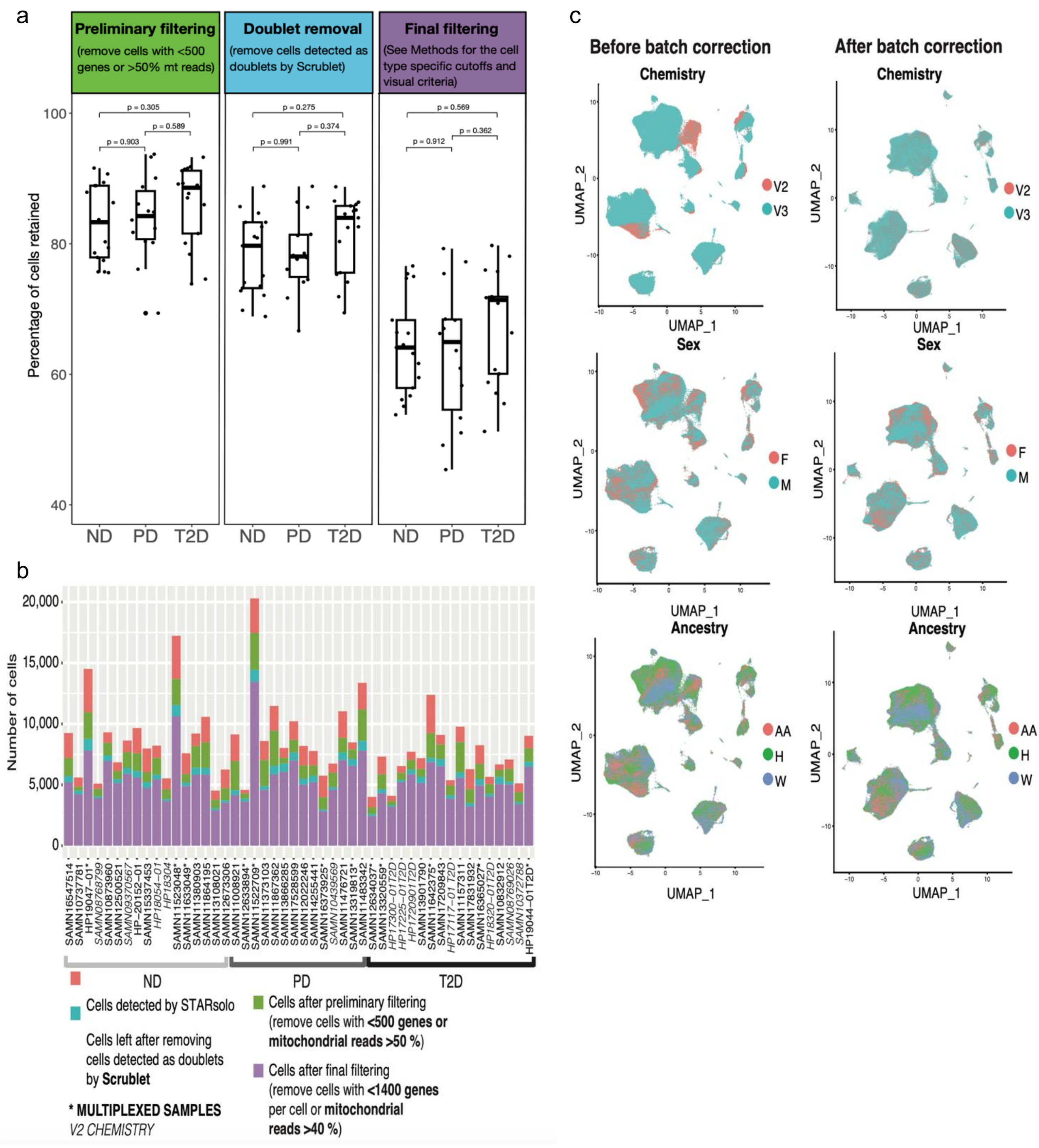

Appendix Figure S1: Quality control measures of single cell transcriptomes from each donor. **(a)** Percent of cells retained (y-axis) for non-diabetic (ND), prediabetic (PD) or type 2 diabetic (T2D) donors after each filtering step (x-axis); each dot represents a donor. Bonferroni-adjusted p-values from Tukey's honest significance test are shown. **(b)** Stacked bar plot indicating number of cells retained (y-axis) per donor (x-axis) after each QC filtering step. Samples with multiplexed runs are typed in bold; 10X sequencing v2 chemistry runs are italicized. **(c)** UMAPs of single cell transcriptomes after preliminary filtering before and after batch correction for sequencing chemistry, sex, and ancestry.

a

	AVERAGE CELLS PER DONOR			MEDIAN CELLS PER DONOR		
	This study	Elgamal	Fold difference	This study	Elgamal	Fold difference
Alpha	1559	772	**2.02**	1281	596	**2.15**
Proliferating Alpha	15	11	**1.36**	10	2	**5.00**
Beta	2063	572	**3.61**	2127	296	**7.19**
Delta	224	56	**4.00**	179	28	**6.39**
Epsilon	4	NR	**NA**	2	NR	**NA**
Gamma	91	NR	**NA**	52	NR	**NA**
Gamma+Epsilon	95	25	**3.80**	54	12	**4.50**
Acinar	279	760	**0.37**	246	354	**0.69**
Ductal	506	339	**1.49**	371	136	**2.73**
Endothelial	91	106	**0.86**	72	42	**1.71**
Mast	7	15	**0.47**	2	11	**0.18**
Quiescent Stellate	227	54	**4.20**	163	28	**5.82**
Activated Stellate	19	124	**0.15**	13	82	**0.16**
**TOTAL***	**5122**	**2957**	**1.73**	**4873**	**2042**	**2.39**

b

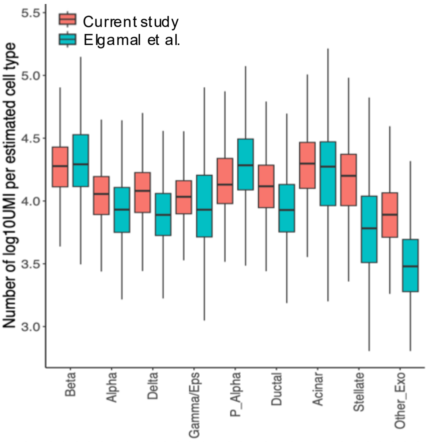

c

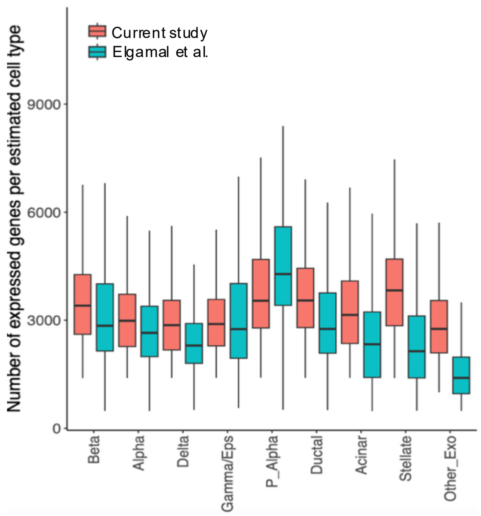

Appendix Figure S2: Comparison of the datasets: current study and Elgamal *et al.* 2023 (*Diabetes*, PMID: 37582230). **(a)** Cell count comparisons. * Total includes all cell types by current study or Elgamal *et al.,* study but not individually listed in this table because of inconsistent annotations (Immune vs. Macrophages) or exclusive inclusion/consideration (‘Alpha+Beta’ by Elgamal study). **(b)** Number of Unique Molecular Identifiers (UMIs) captured per cell type in the current study vs. Elgamal *et al.*, shown on a $\log_{10}$ scale. Boxes show the interquartile range (25th-75th percentiles) with the median indicated. **(c)** Distribution of number of expressed genes in scRNA-seq profiles. Box plots denote the 25th, median, and 75th percentile values of distributions in current study vs. Elgamal *et al.,* study.

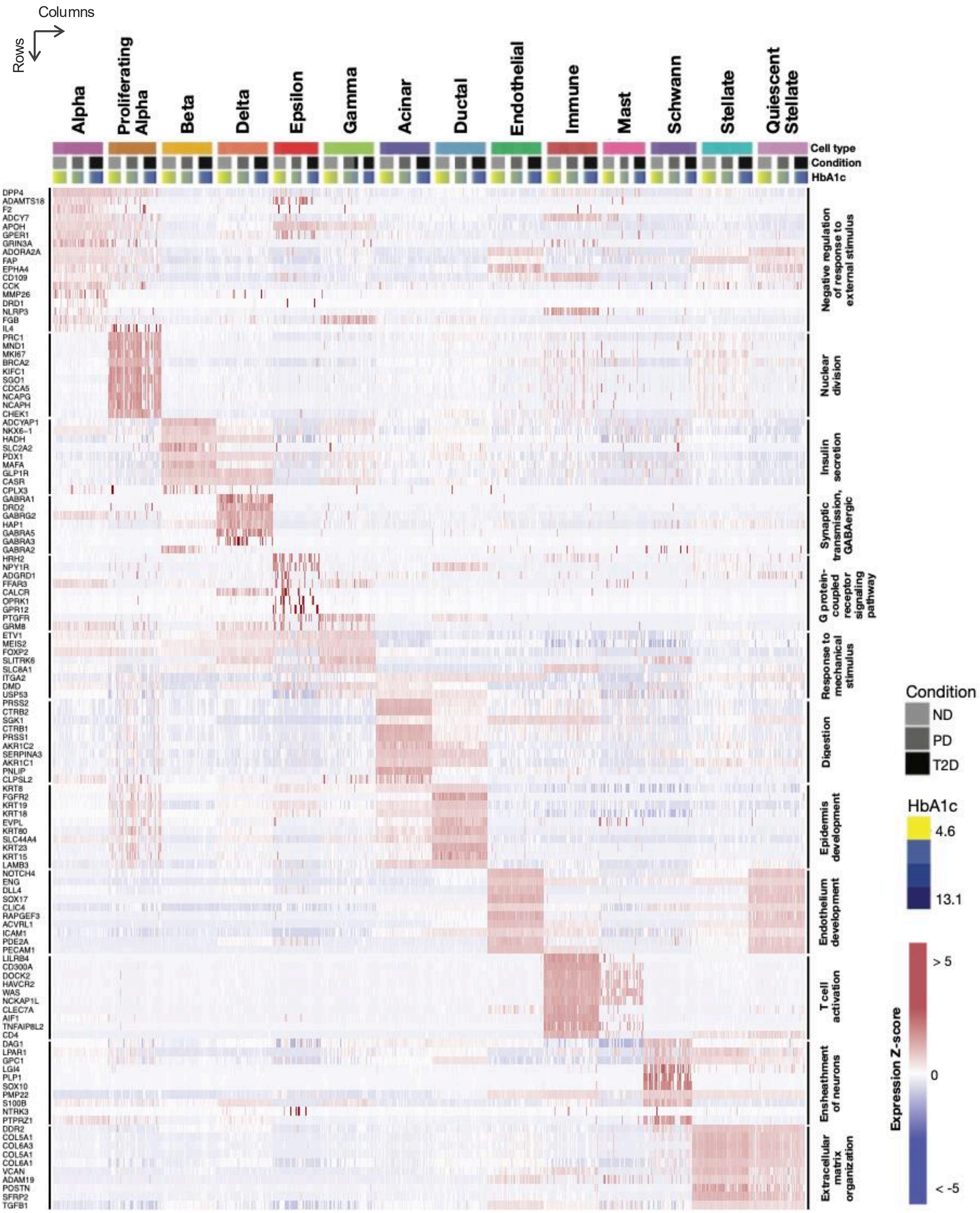

Appendix Figure S3: **(a)** Heatmap of aggregated (pseudobulk) marker gene expression (left; rows) representing enriched GO terms (right) for each islet cell type (columns). Individual islet donors are grouped by glycemic status (grayscale) and sorted from lowest to highest reported HbA1c levels (yellow-blue gradient) for each cell type and glycemic status shown . ND: Non-diabetic, PD: prediabetic, T2D: Type 2 diabetic. Full set of enriched marker genes and GO terms for each cell type are provided in Dataset EV2 and EV3.

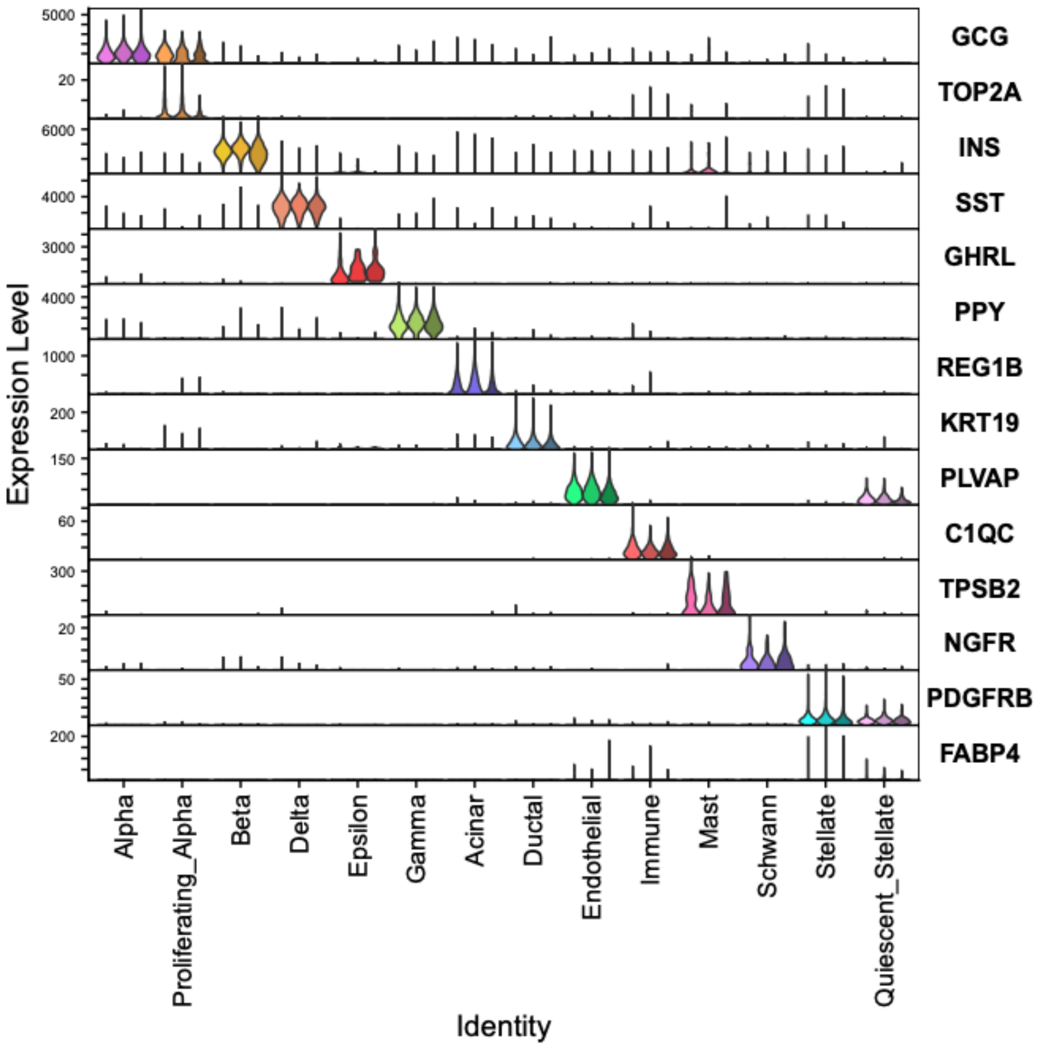

Appendix Figure S3: **(b)** Violin plots showing expression (counts per million) of key marker genes in various islet cell types. First violin in each cell type denotes ND donors, second: PD donors and third: T2D donors.

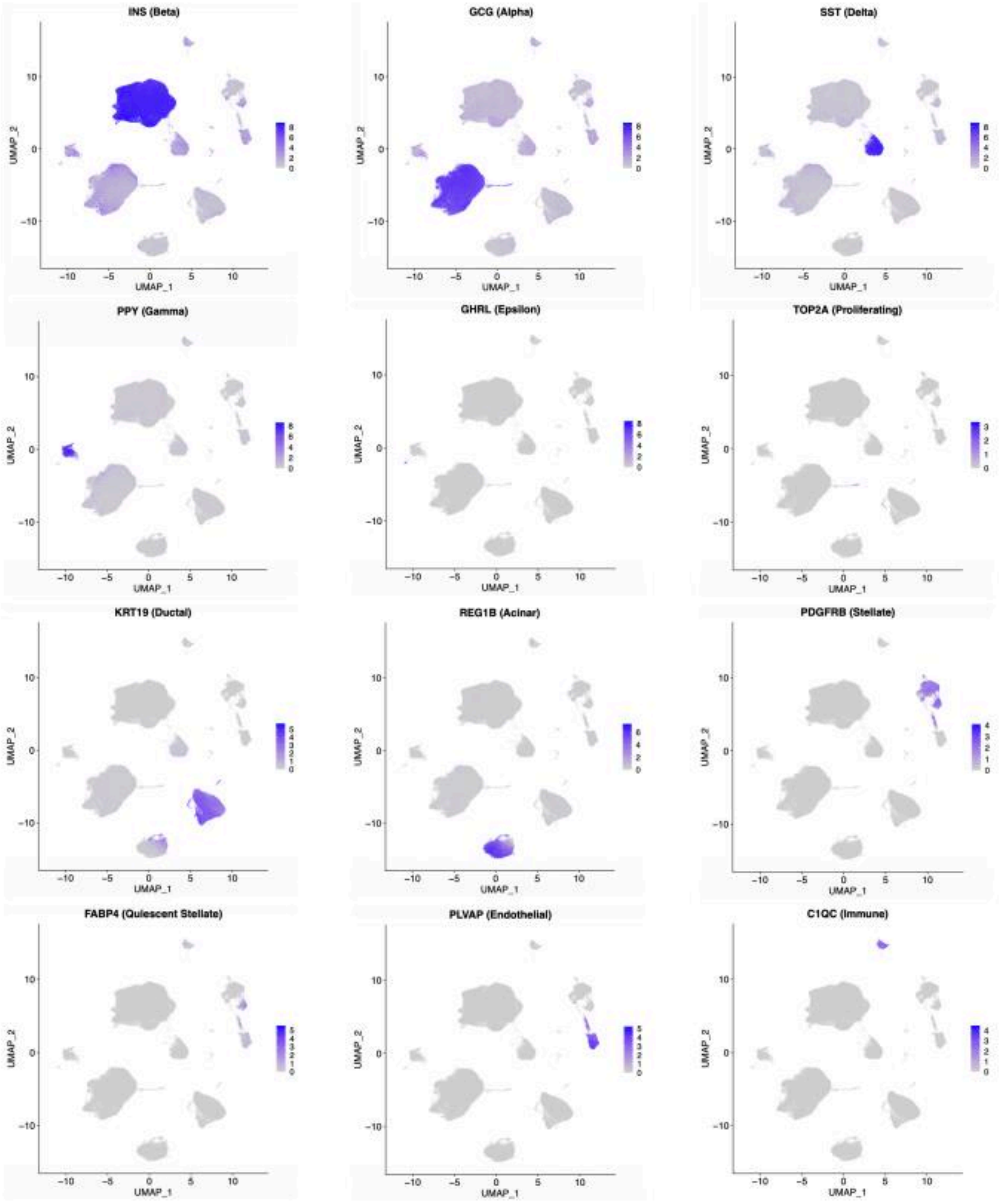

Appendix Figure S3: **(c)** UMAPs showing marker gene expression in each cell type for all donors.

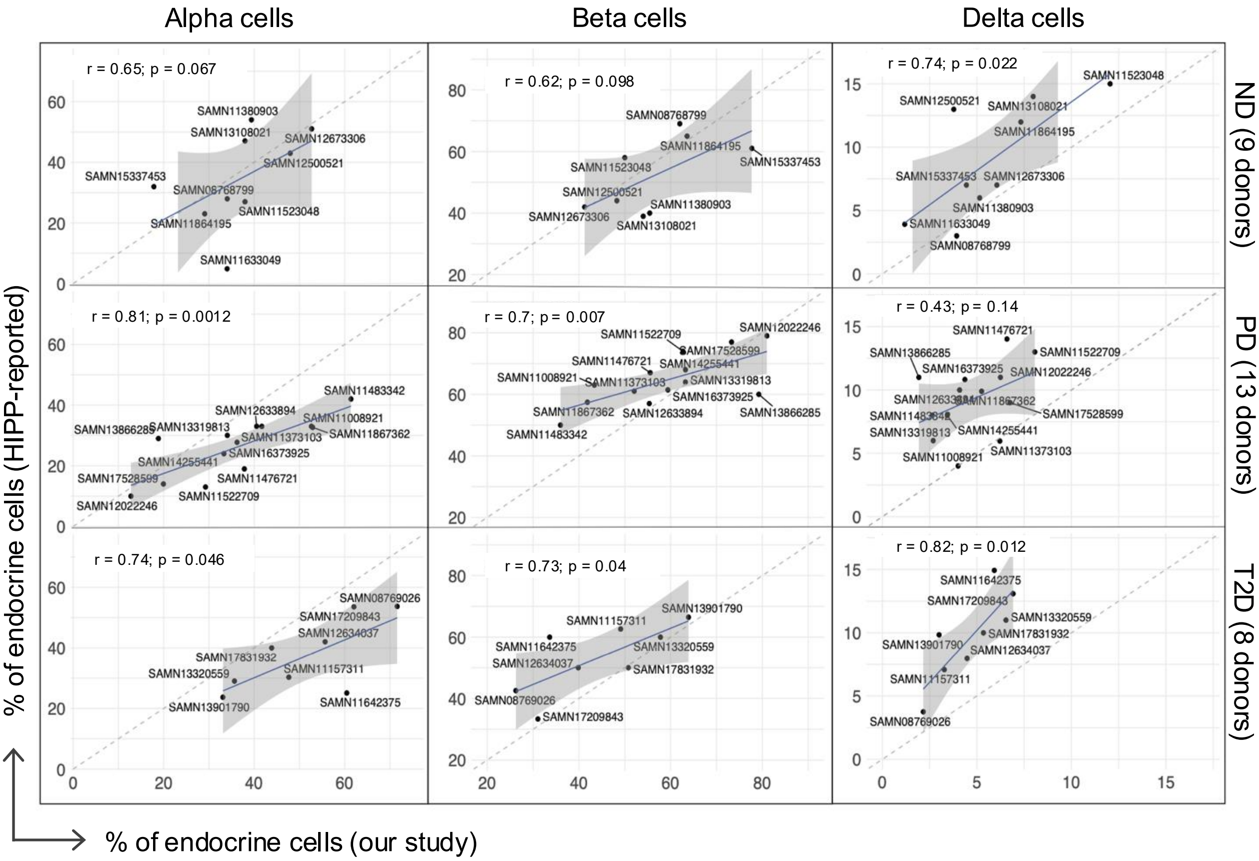

Appendix Figure S4: Scatter plots of spearman correlation (r) between alpha, beta, and delta cell endocrine proportions reported by the the Human Islet Phenotyping Program (HIPP, y-axis) for 9 Non-diabetic (ND), 13 prediabetic (PD), and 8 type 2 diabetic donors and as detected by our scRNA-seq counts (x-axis) from the corresponding donors. The identity line (dashed) and the line of fitted linear regression model (solid blue) are shown.

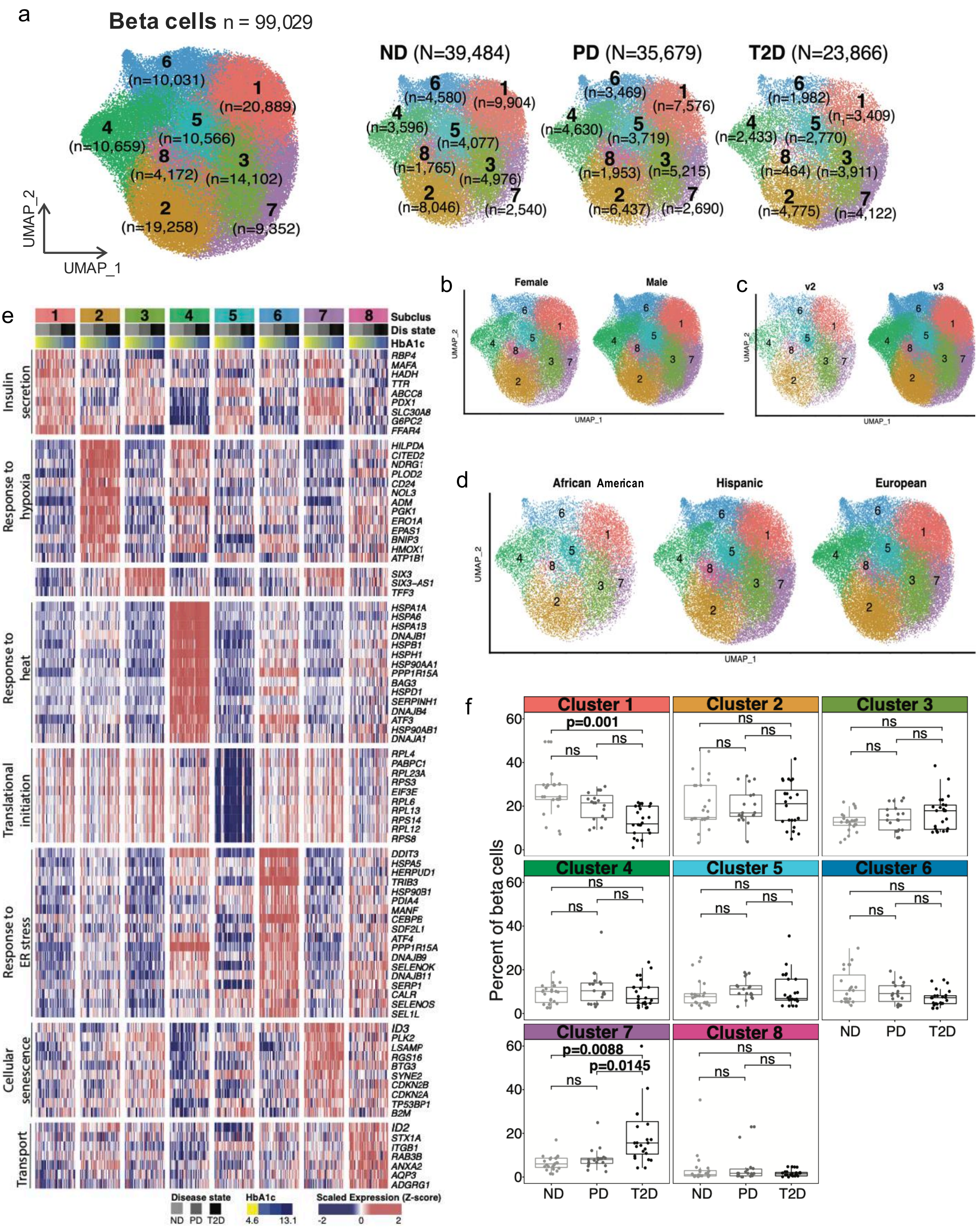

Appendix Figure S5: UMAPs of beta-cell subpopulations shown for each glycemic status (**a**), sex (**b**), scRNA sequencing chemistry (**c**; v2 or v3), and self-reported ancestry (**d**; European, African American and Hispanic). Number of cells per cluster (n) is indicated in parentheses. (**e**) Heatmap of normalized marker gene expression in beta-cell subpopulations. (**f**) Putative β-cell subpopulation proportions in non-diabetic (ND; n = 17), prediabetic (PD; n = 14) and type 2 diabetic (T2D; n = 17) donors. Individual dots display per-donor proportions in each group. P-values between groups are calculated from Tukey's honestly significance test, adjusting for Bonferroni correction.

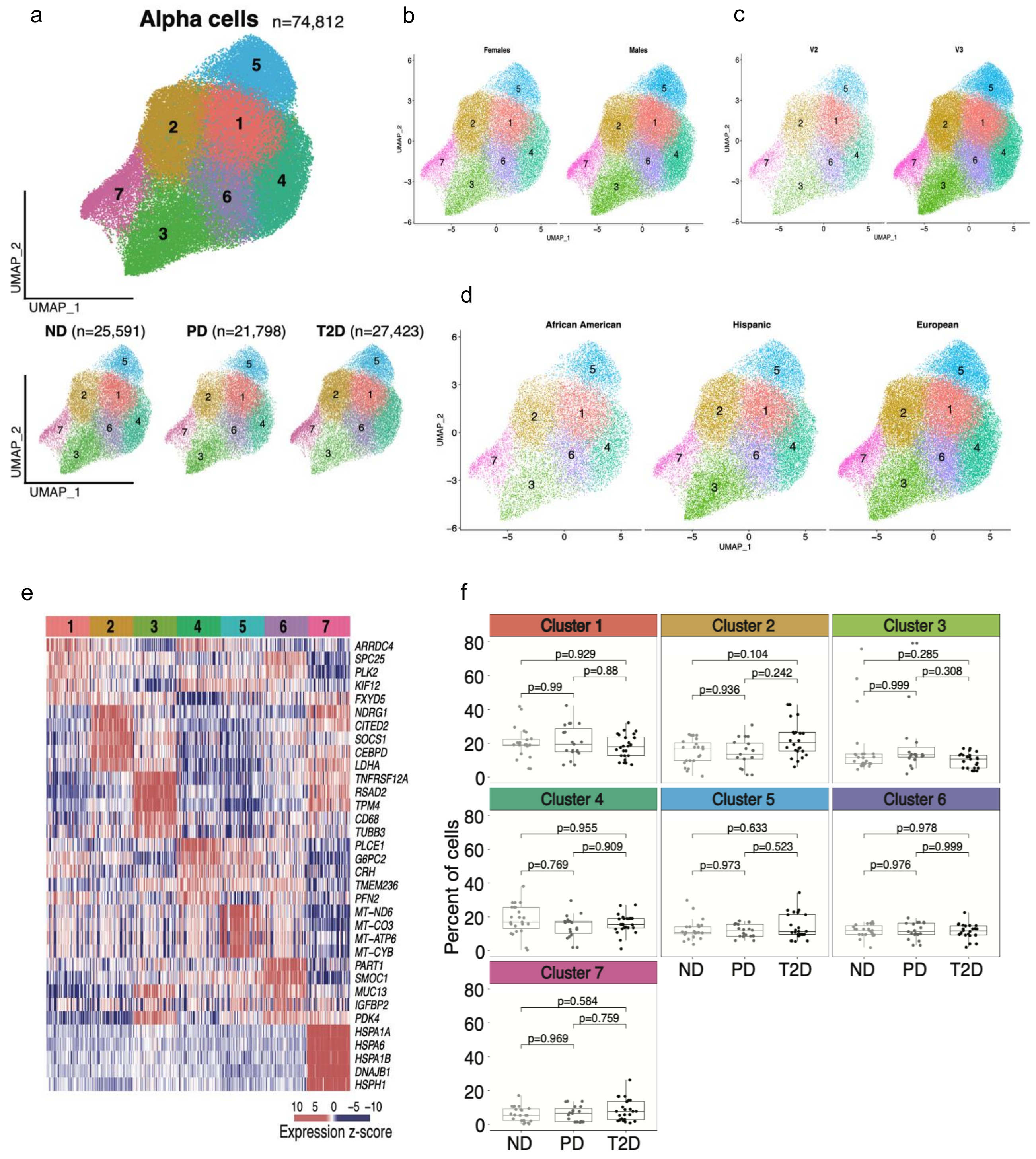

Appendix Figure S6: UMAPs of alpha-cell subpopulations shown for each glycemic status (**a**), sex (**b**), scRNA sequencing chemistry (**c**; v2 or v3**)**, and self-reported ancestry (**d**; European, African American and Hispanic). Number of cells per cluster (n) is indicated in parentheses. (**e**) Heatmap of normalized marker gene expression in alpha-cell subpopulations. (**f**) Putative α-cell subpopulation proportions in non-diabetic (ND; n = 17), prediabetic (PD; n = 14) and type 2 diabetic (T2D; n = 17) donors. Individual dots display per-donor proportions in each group. P-values between groups are calculated from Tukey's honestly significance test, adjusting for Bonferroni correction.

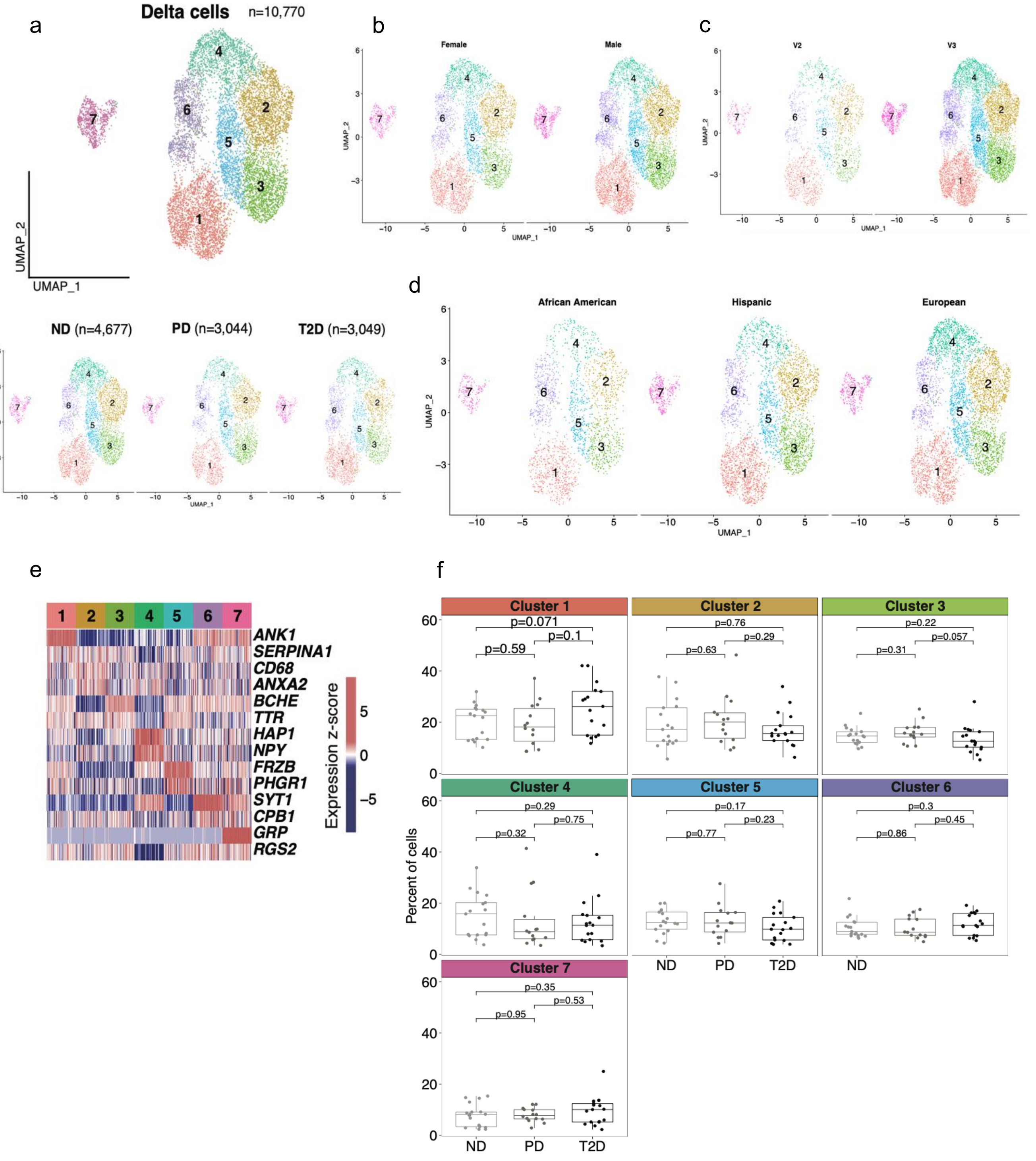

Appendix Figure S7: UMAPs of delta-cell subpopulations shown for each glycemic status (**a**), sex (**b**), scRNA sequencing chemistry (**c**; v2 or v3**)**, and self-reported ancestry (**d**; European, African American and Hispanic). Number of cells per cluster (n) is indicated in parentheses. (**e**) Heatmap of normalized marker gene expression in delta-cell subpopulations. (**f**) Putative **δ**-cell subpopulation proportions in non-diabetic (ND; n = 17), prediabetic (PD; n = 14) and type 2 diabetic (T2D; n = 17) donors. Individual dots display per-donor proportions in each group. P-values between groups are calculated from Tukey's honestly significance test, adjusting for Bonferroni correction.

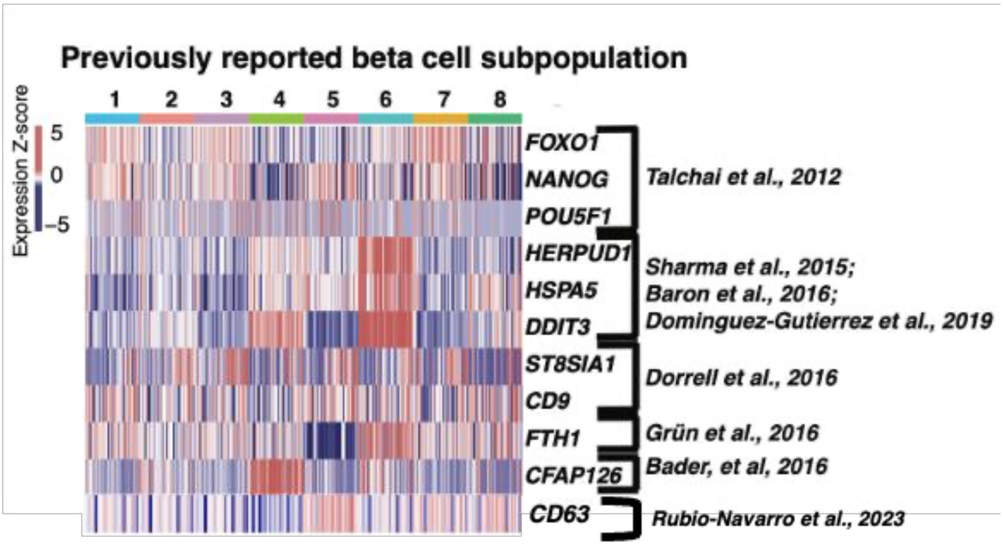

Appendix Figure S8: Heatmap of scaled expression of previously reported beta-cell subpopulation marker genes (PMID: 27399229, PMID: 26389675, PMID: 27667365, PMID: 31500834, PMID: 27398620, PMID: 36928765, PMID: 22980982 and PMID: 27345837) in this study.

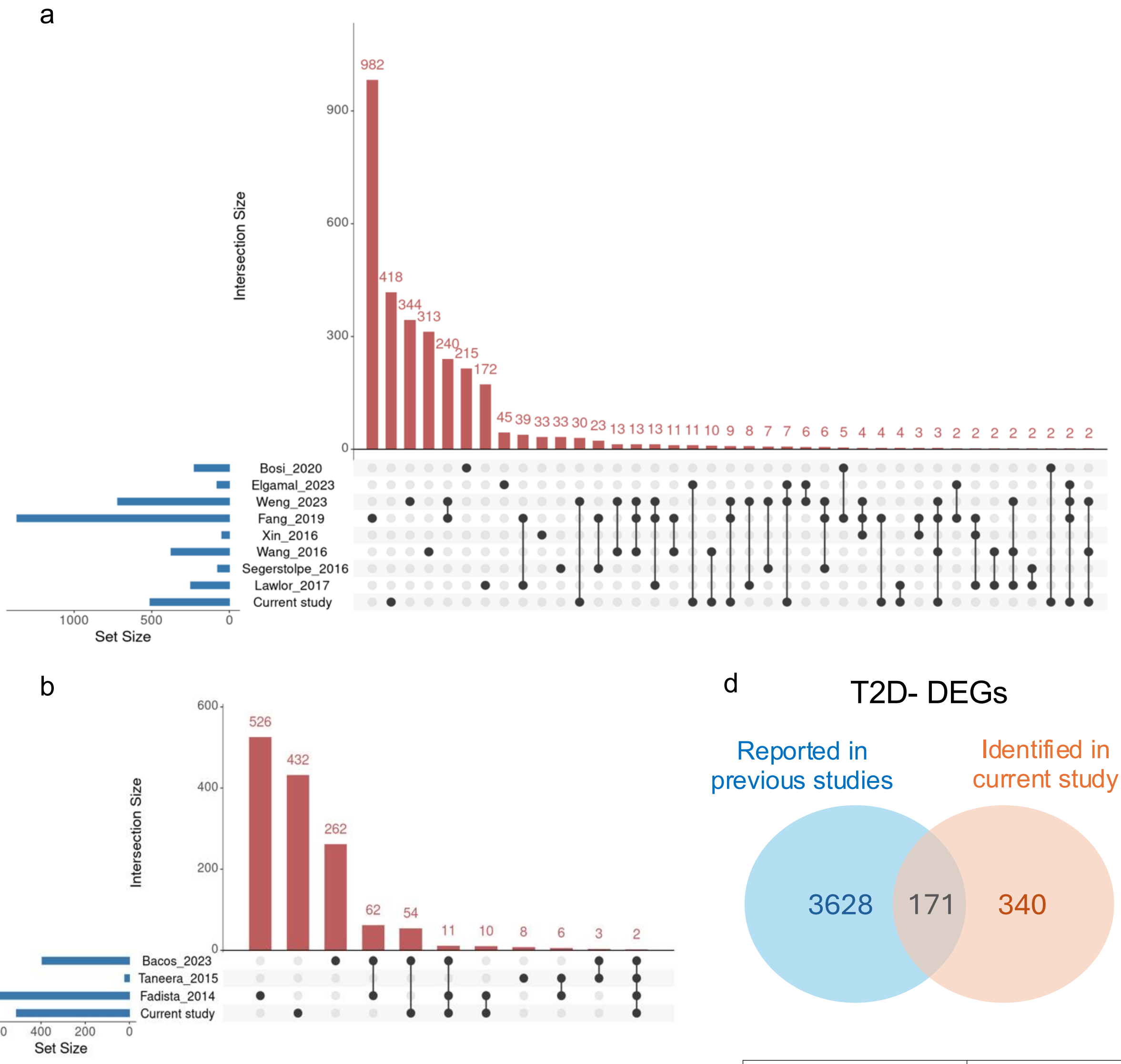

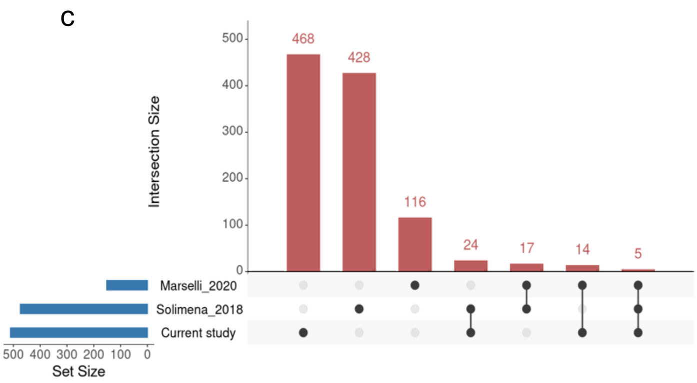

Reported studies	PubMed IDs
Bacos_2023	36656641
Bosi_2020	33575641
Elgamal_2023	37582230
Fadista_2014	25201977
Fang_2019	30865899
Lawlor_2017	27864352
Marselli_2020	33264613
Segerstolpe_2016	27667667
Solimena_2018	29185012
Taneera_2015	25489054
Wang_2016	27364731
Weng_2023	37669939
Xin_2016	27667665

Appendix Figure S9: UpSet plots showing overlap between the identified T2D vs ND β-cell differentially expressed genes (DEGs) with previously reported DEGs – β-cell scRNA-seq **(a)**, islet RNA-seq **(b),** and sorted β-cell RNA-seq **(c)**. Overlaps containing at least 2 common genes are shown. **(d)** Venn diagram showing our replication of 171 previously reported genes and identification of 340 novel T2D-DEGs in current study.

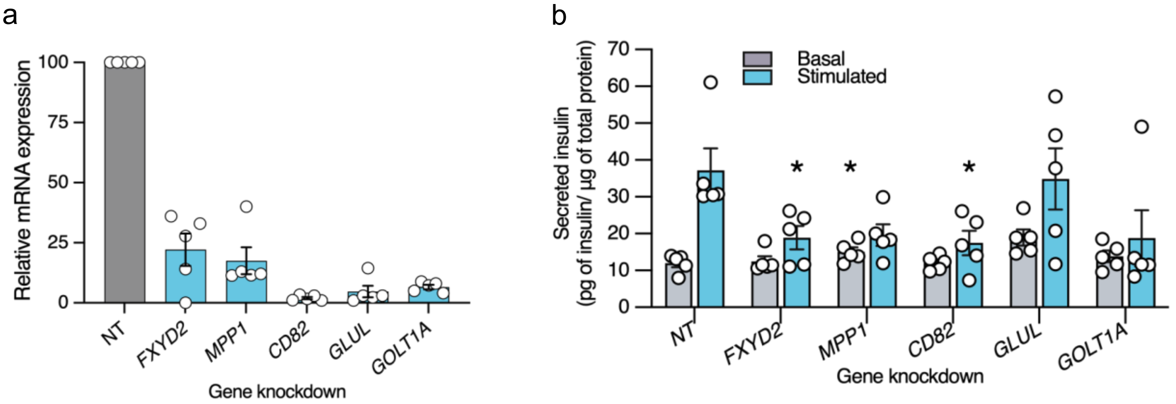

Appendix Figure S10: **(a)** shRNA mediated knockdown of selected T2D-downregulated β-cell genes in human EndoC-βH3 cells. Gene expression is shown relative to that in non-targeting (NT) shRNA transduced cells. Data was plotted as mean ± standard error of the means (s.e.m.) from 5 biological replicates. **(b)** Basal (gray; 0mM glucose) or glucose-stimulated (blue; 20mM glucose) insulin secretion in human EndoC-βH3 cells following knockdown of selected target genes. Data was plotted as mean ± s.e.m. from 5 biological replicates, each represented by dots. Significance was calculated relative to corresponding conditions for NT control cells using paired Student's t-test (two-tailed, paired) where $*p < 0.05$.

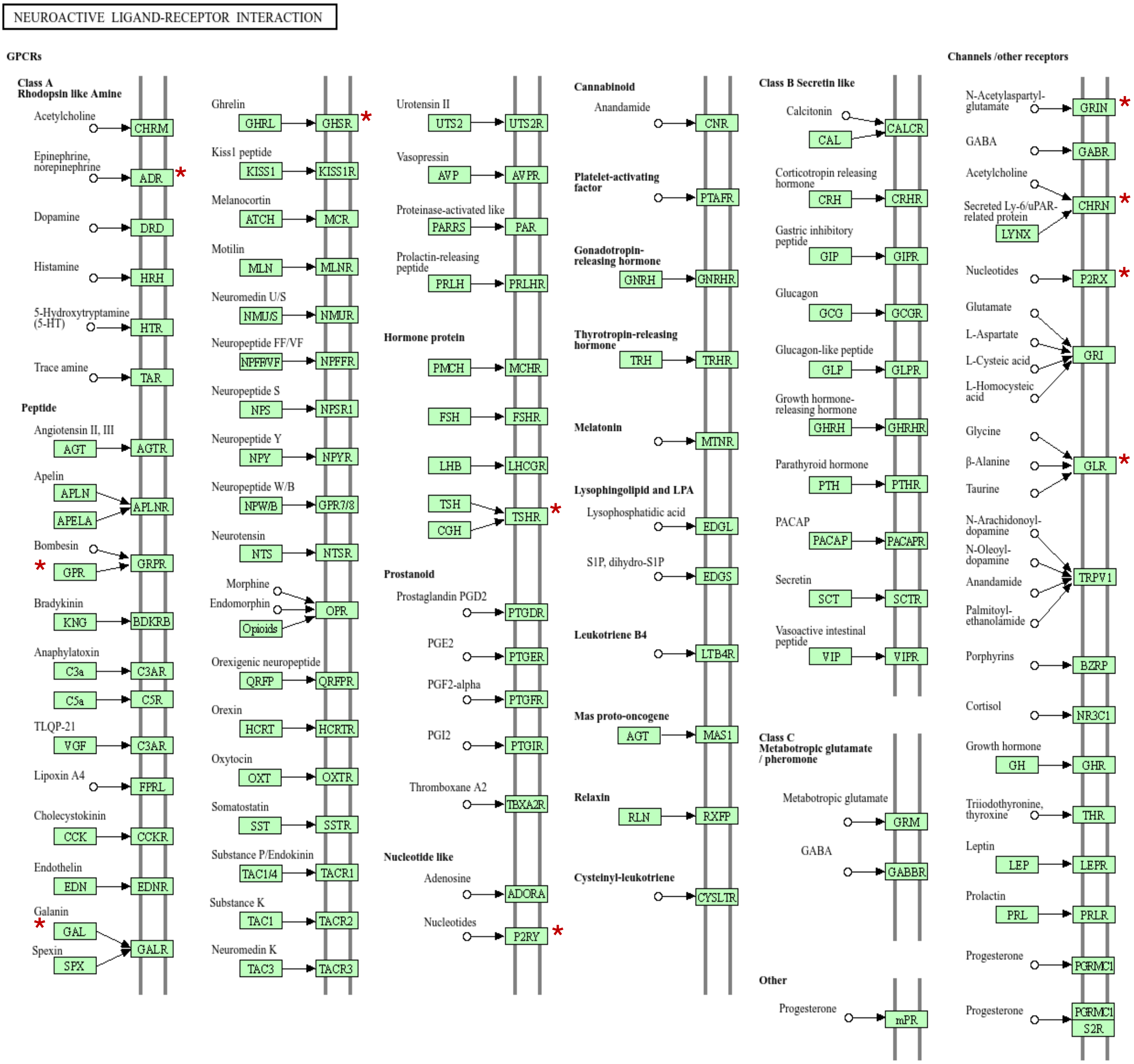

Appendix Figure S11: Primary pathways associated with T2D β-cell differentially expressed genes (DEGs). ‘Neuroactive ligand receptor interaction’ enrichment in upregulated genes.
* marks identified T2D β-cell DEGs in the pathways. Image sources: KEGG and Wikipathways.

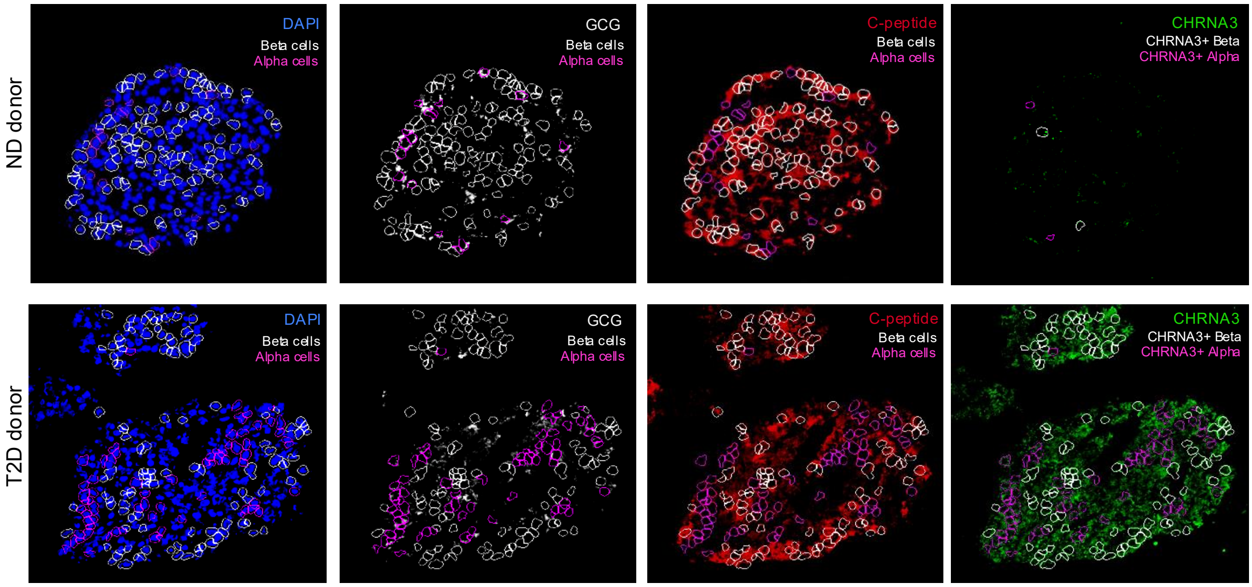

Appendix Figure S12: Representative immunofluorescence (IF) images from figure 3b showing the spatial distribution and detection of CHRNA3$^+$ β- and α-cells. Cell segmentation was performed using CellProfiler, with DAPI staining employed to identify nuclei as primary objects. Nuclei were expanded to approximate whole cell boundaries. Donor-specific mean fluorescence intensities were used to classify β- and α-cells, enabling exclusion of unresolved cells. CHRNA3$^+$ cells were subsequently identified within the resolved populations based on donor specific mean intensity threshold. Magenta outlined cells: α-cells; White outlined cells: β-cells.

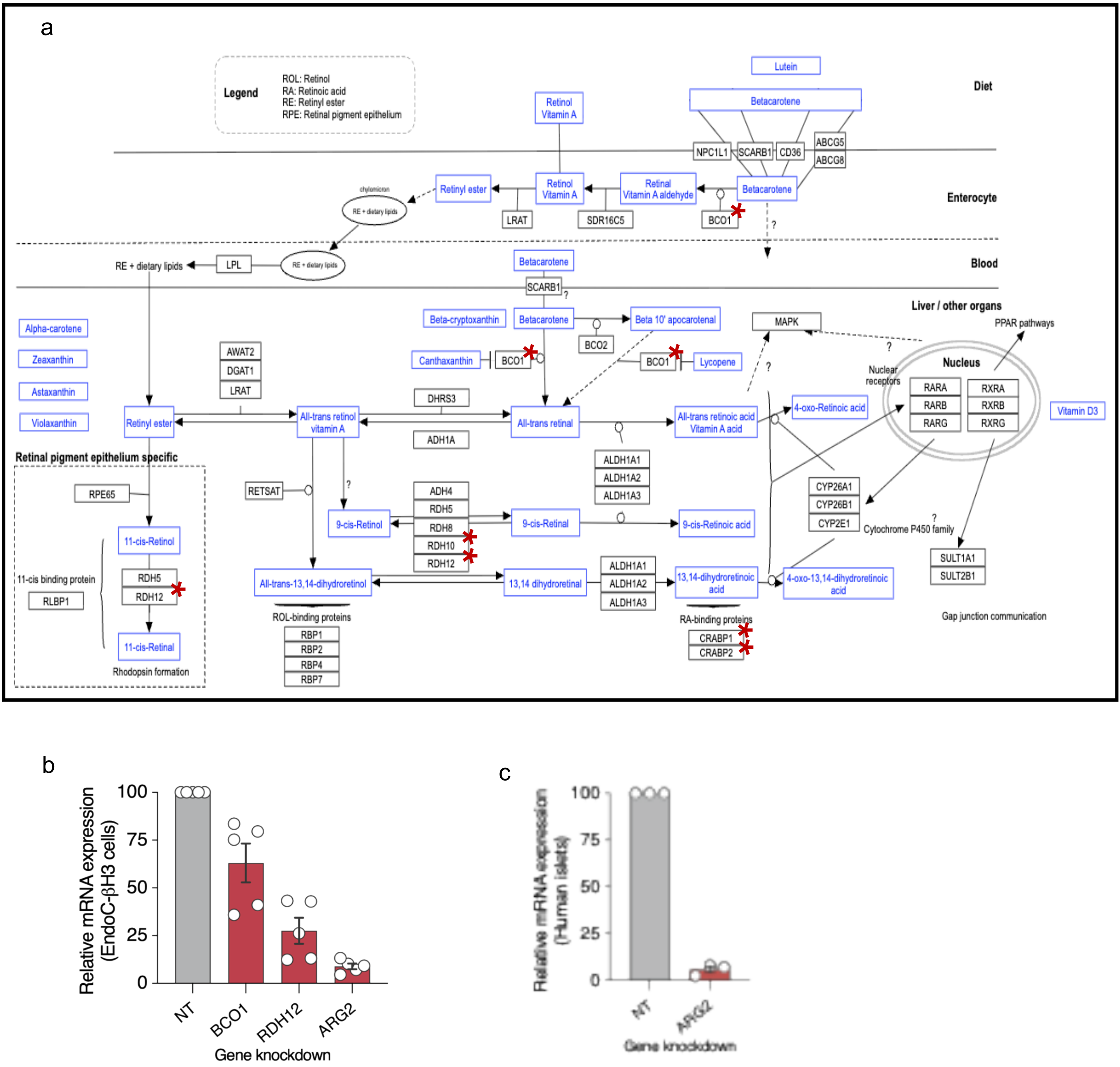

Appendix Figure S13: **(a)** 'Vitamin A and carotenoid metabolism' in downregulated genes. * marks identified T2D β-cell DEGs in the pathways. Image sources: KEGG and WikiPathways. **(b)** Expression of vitamin A metabolism pathway genes (*BCO1* and *RDH12*) and the downstream target gene (*ARG2*) in human EndoC-βH3 cells following their shRNA-mediated knockdown, shown relative to the non-targeting (NT) control. Data was plotted as mean mean ± s.e.m. from 5 biological replicates. **(c)** Expression of *ARG2* in β-cell enriched human pseudoislets following shRNA-mediated knockdown. Data was plotted as mean ± s.e.m. for islets from 3 non-diabetic (ND) donors.

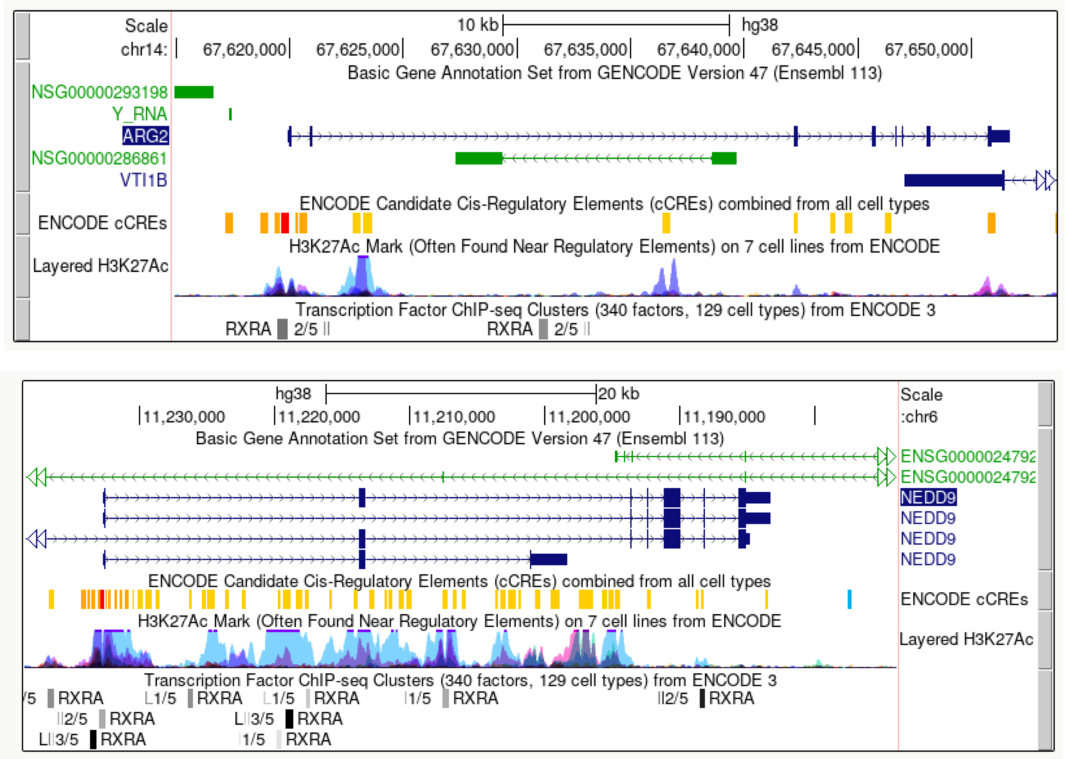

Appendix Figure S14: Genomic locus snapshots of β-cell death-related downregulated DEGs, showing the presence of retinoic acid X receptor (RXRA) binding elements. These elements regulate the expression of RA target genes. Data source: ENCODE TF ChIp-seq, UCSC Genome Browser.

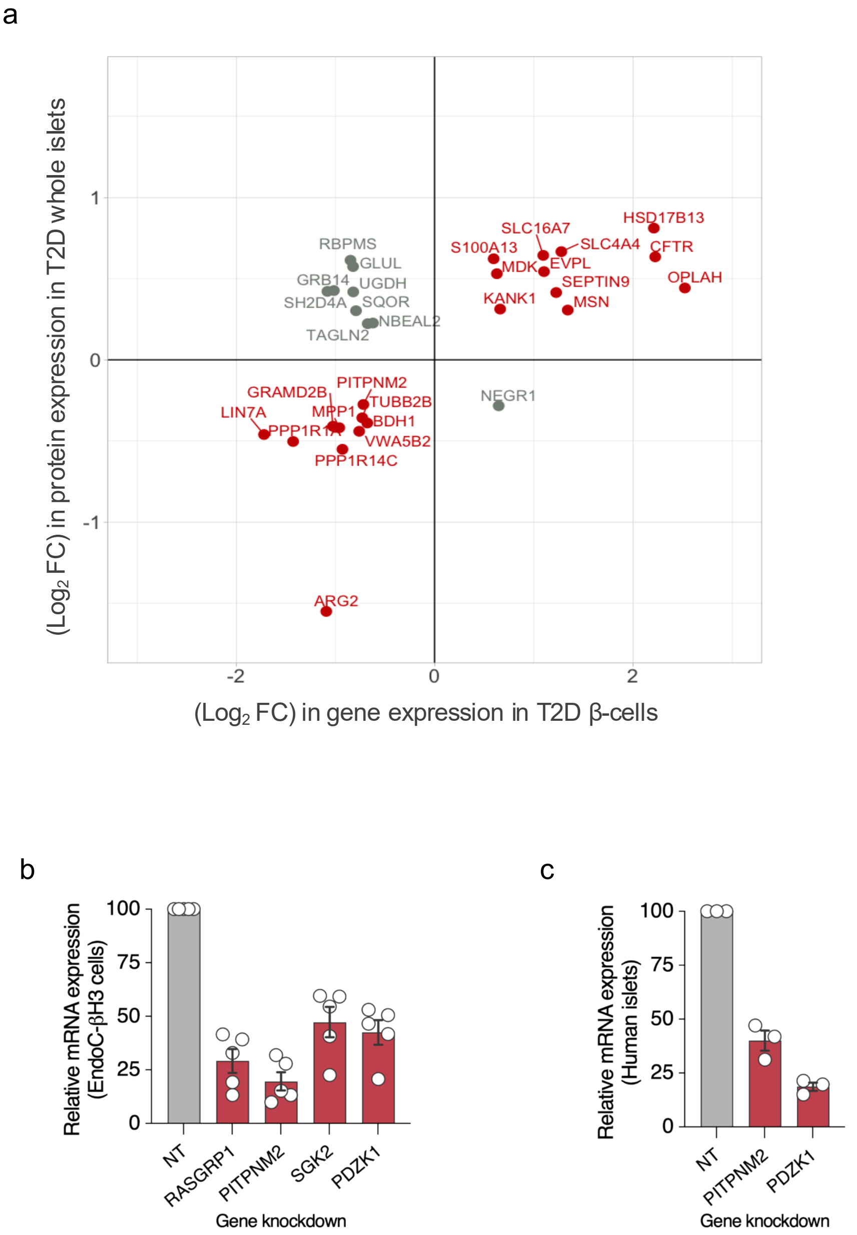

Appendix Figure S15: **(a)** Comparison of protein expression and gene expression in human islets. Log_2 fold change (FC) protein expression in whole T2D islets (*https://www.humanislets.com/#/* (PMID: 39357523), y-axis) vs. Log_2 FC in gene expression for T2D β-cell differentially expressed genes (DEGs, x-axis) from this study. Red denotes genes with consistent T2D effects on protein and gene expression. Upper right quadrant genes are concordantly upregulated; lower left quadrant genes are concordantly downregulated. Gray denotes genes with opposite islet protein expression vs. T2D β-cell differential expression. **(b)** Expression of T2D genetic targets in human EndoC-βH3 cells following their shRNA-mediated knockdown, shown relative to the non-targeting (NT) control. Data was plotted as mean ± s.e.m. from 5 biological replicates. **(c)** Relative mRNA levels of *PITPNM2* and *PDZK1* in β-cell enriched human pseudoislets following shRNA-mediated knockdown. Data was plotted as mean ± s.e.m. for islets from 3 non-diabetic (ND) donors.

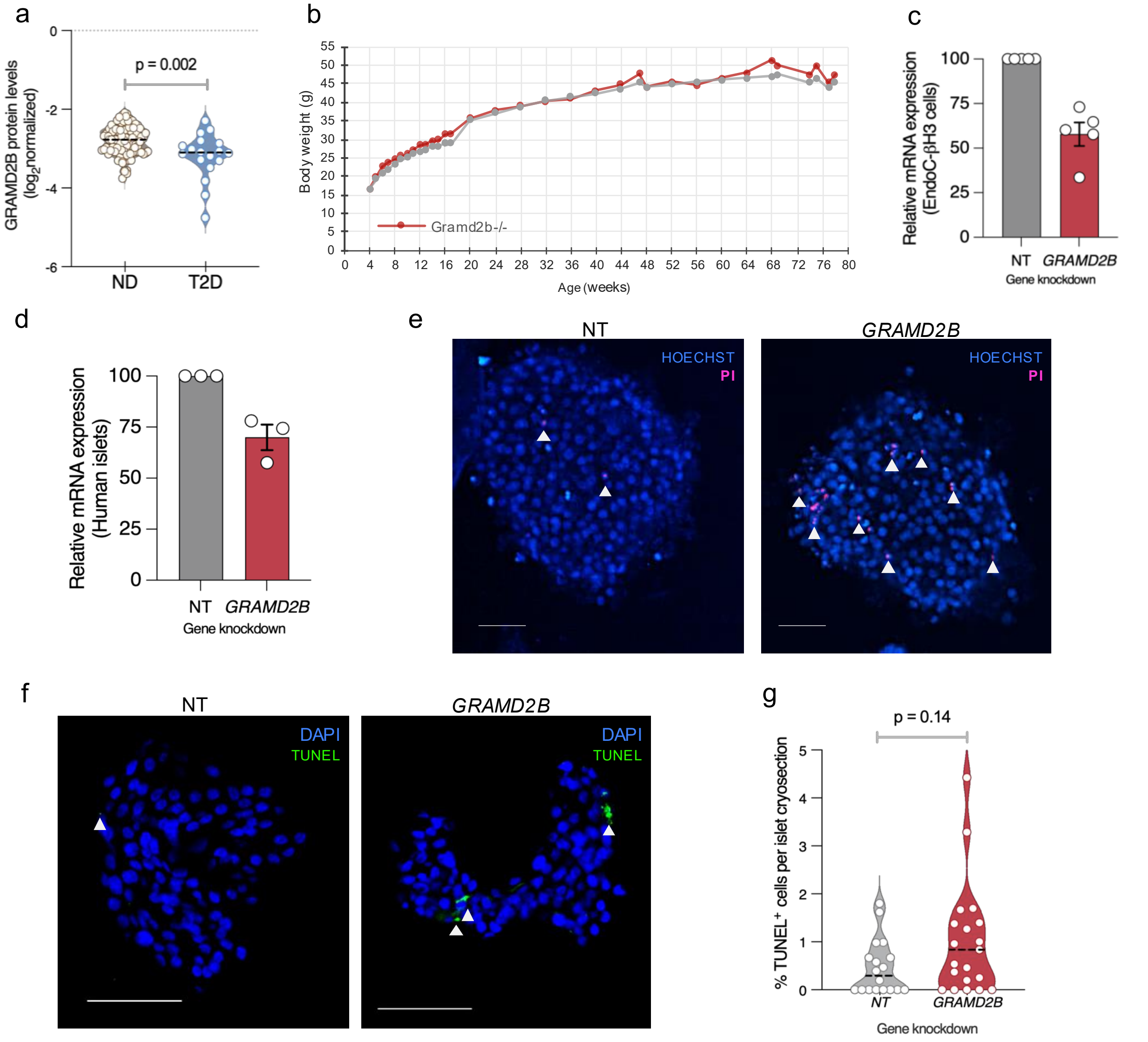

Appendix Figure S16: **(a)** GRAMD2B protein levels in ND vs. T2D islets in humanislets.com cohort (PMID: 39357523). **(b)** Growth curve showing the average bodyweight measures of WT and *Gramd2b* knockout male mice from 4 to 78 weeks of age (https://www.mousephenotype.org/). **(c)** Relative *GRAMD2B* expression in human EndoC-βH3 β-cells following shRNA-mediated knockdown compared to non-targeting (NT) control. Data was plotted as mean ± s.e.m. from 5 biological replicates. **(d)** *GRAMD2B* expression in human β-pseudoislets after shRNA-mediated knockdown relative to the NT shRNA control islets. Data was plotted as mean ± s.e.m. for islets from 3 non-diabetic (ND) donors. **(e)** Representative live images of one β-cell enriched human pseudoislet after shRNA-mediated knockdown of *GRAMD2B* (right) versus NT (left) stained with Hoechst and propidium iodide (PI, pink). Scale bar for all images = 50µm. White arrows indicate examples of PI^+ cells. **(f)** Representative immunofluorescence cryosection images of human β-pseudoislets following TUNEL staining (green) after *GRAMD2B* knockdown, magnification=40x. White arrows show examples of $TUNEL^+$ cells. Scale bar for all images = 50µm. **(g)** Violin plot of the distribution of $TUNEL^+$ cells per islet cryosection in NT or *GRAMD2B* shRNA-transduced β-pseudoislets reaggregated from native islets of 3 ND donors. Measurements were obtained from an average of 7 sections from each of three islet donors. The black dashed line indicates the median. One datapoint is higher than the plotted y-axis limit. P value was calculated from a mixed-effects linear regression model with donor ID as the random effect.
