# Supplementary figures and images for: Deep single-cell decoding of human pancreatic islets reveals T2D β-cell gene expression defects
Source: EMBO J. 2026 Apr 15;45(11):3978–4005. doi: 10.1038/s44318-026-00744-w (PMC13226668; doi:10.1038/s44318-026-00744-w)

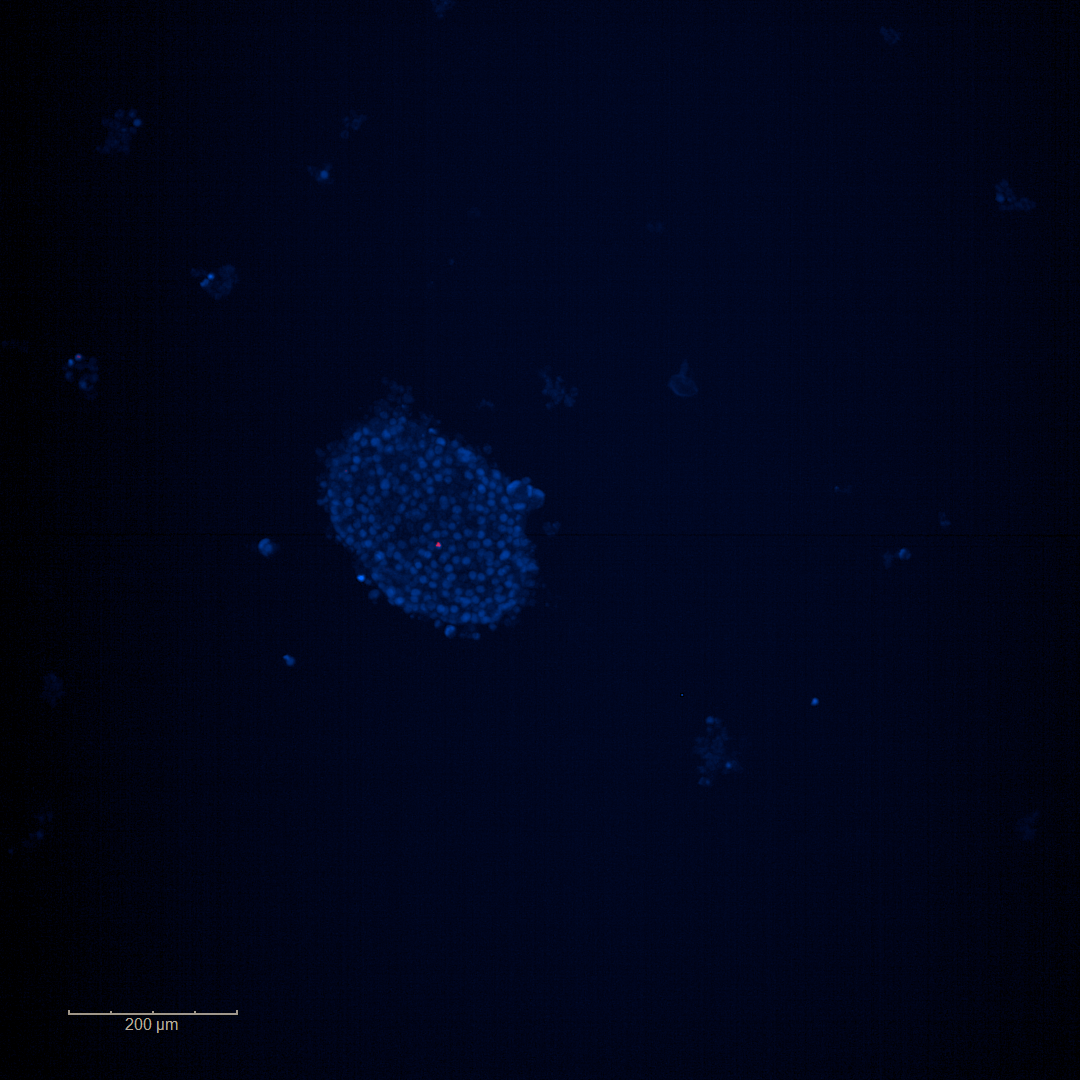

Supplement: Supplementary file 21 — Source data Fig. 3 [file 44318_2026_744_MOESM21_ESM.zip › Figure 3/I/islet227_CTR_B6_PI.tiff]

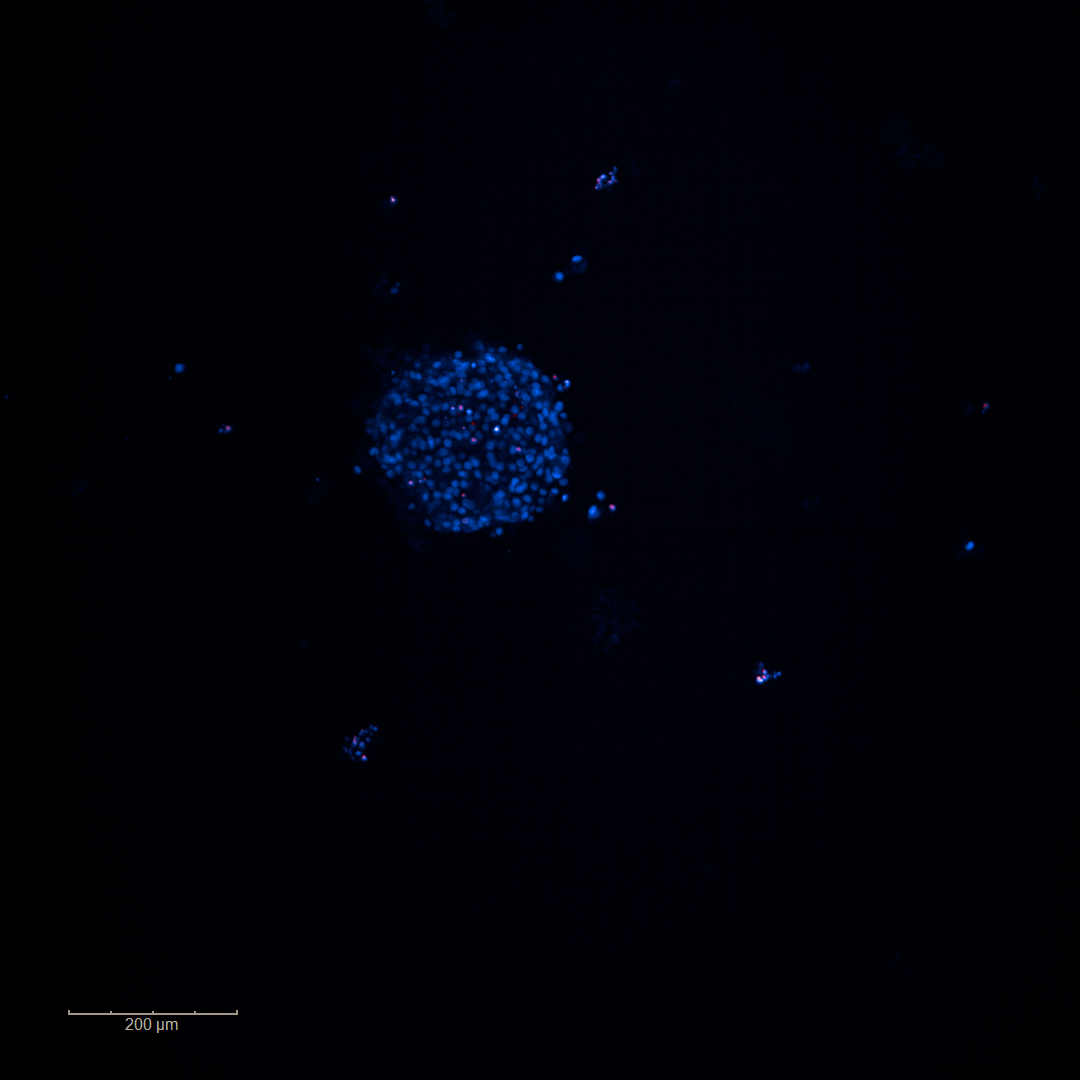

Supplement: Supplementary file 21 — Source data Fig. 3 [file 44318_2026_744_MOESM21_ESM.zip › Figure 3/I/islet227_ARG2_E12.tiff]

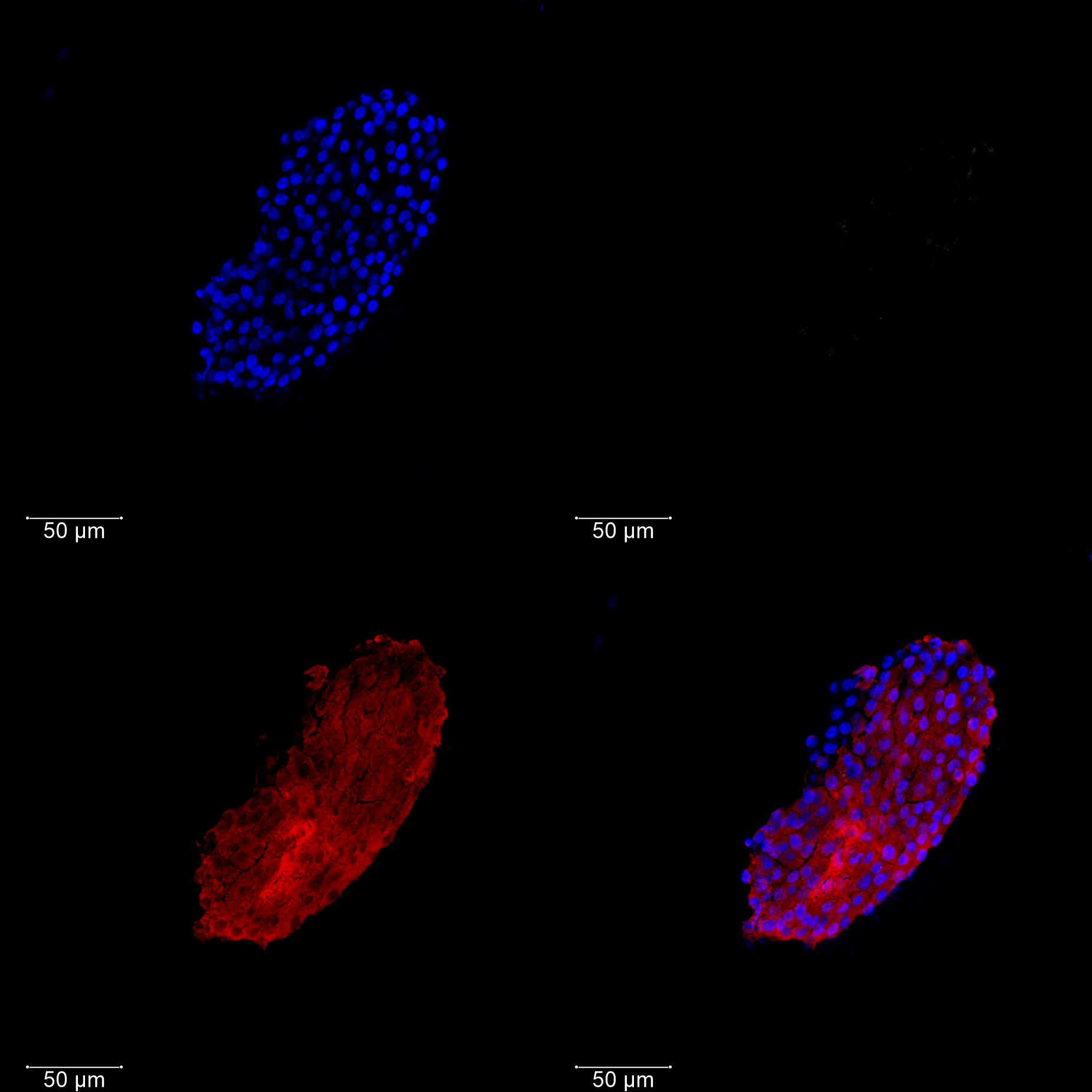

Supplement: Supplementary file 21 — Source data Fig. 3 [file 44318_2026_744_MOESM21_ESM.zip › Figure 3/H/islet227_CTR_Snapshot all.tif]

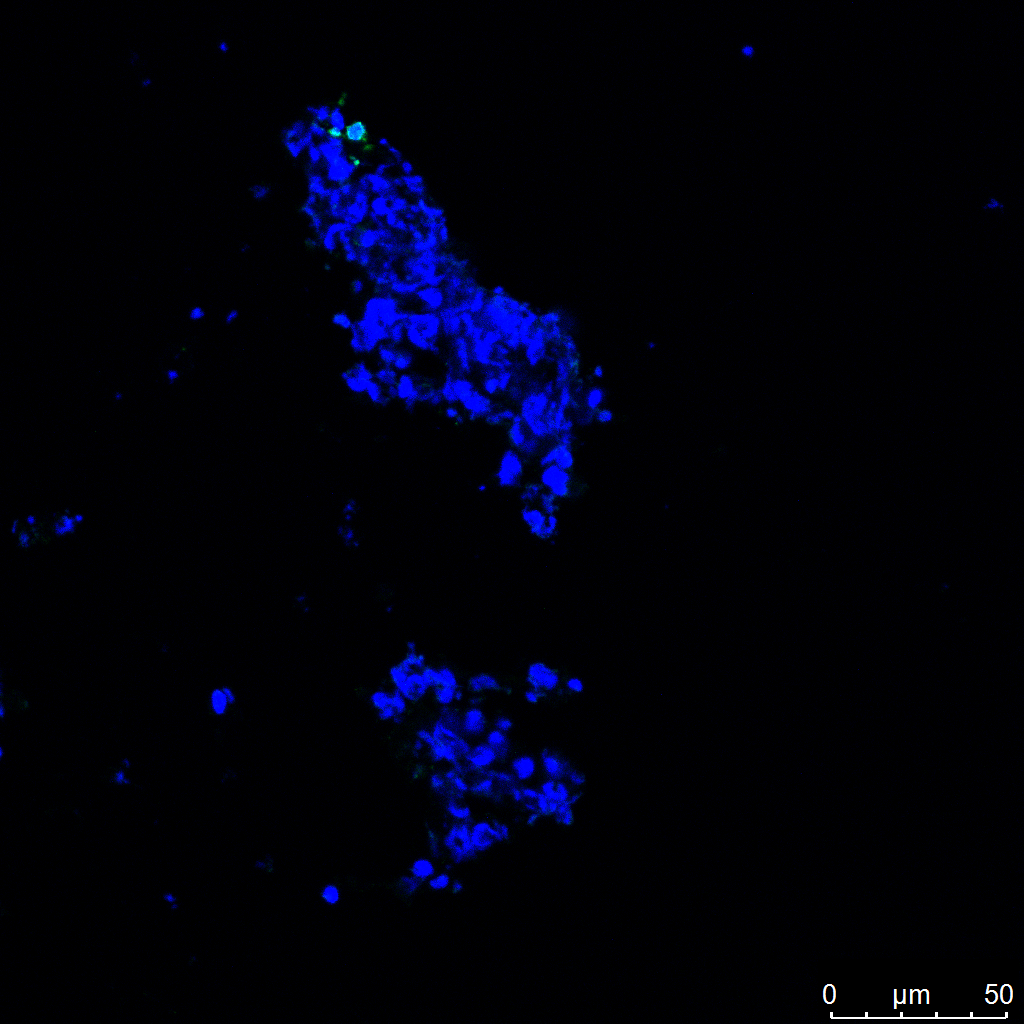

Supplement: Supplementary file 21 — Source data Fig. 3 [file 44318_2026_744_MOESM21_ESM.zip › Figure 3/K/Islet227_TUNEL_ARG2_2.tif]

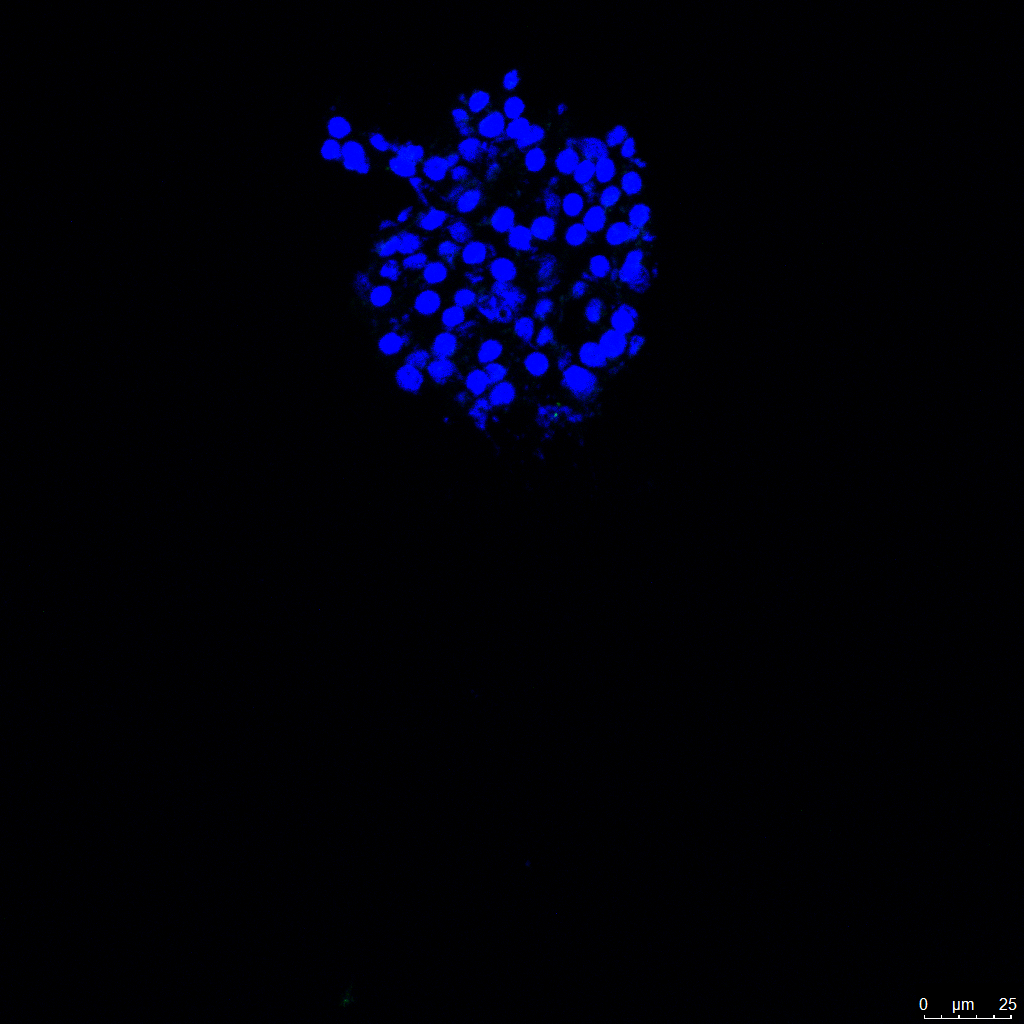

Supplement: Supplementary file 21 — Source data Fig. 3 [file 44318_2026_744_MOESM21_ESM.zip › Figure 3/K/Islet227_TUNEL_CTR_4.tif]

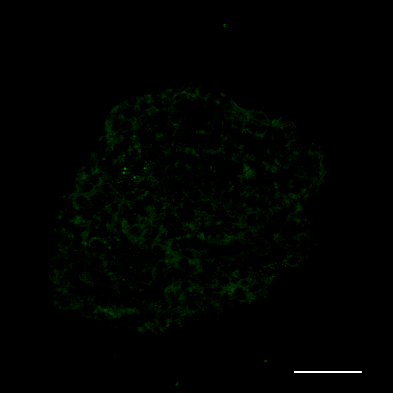

Supplement: Supplementary file 21 — Source data Fig. 3 [file 44318_2026_744_MOESM21_ESM.zip › Figure 3/B/FIGURE 3B/bandek_20250618_ND_islet34_CHRNA3_40x_4_chrna3.tif]

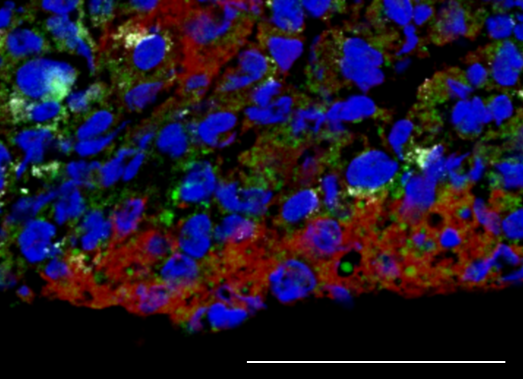

Supplement: Supplementary file 21 — Source data Fig. 3 [file 44318_2026_744_MOESM21_ESM.zip › Figure 3/B/FIGURE 3B/bandek_20250618_T2D_islet71_CHRNA3_40x_1_zoom.tif]

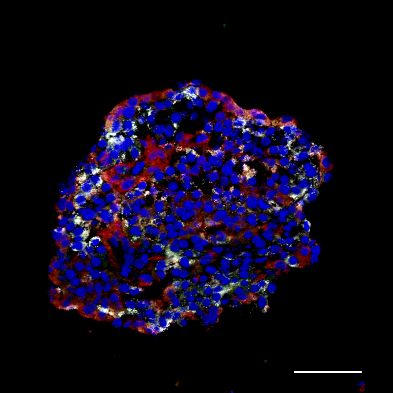

Supplement: Supplementary file 21 — Source data Fig. 3 [file 44318_2026_744_MOESM21_ESM.zip › Figure 3/B/FIGURE 3B/bandek_20250618_ND_islet34_CHRNA3_40x_4_all.tif]

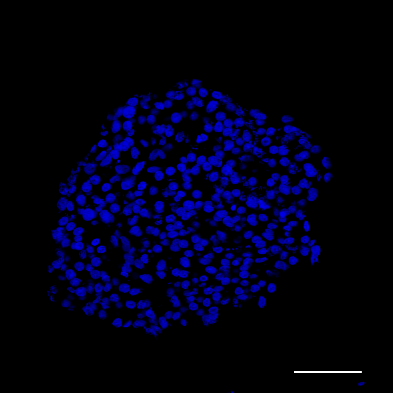

Supplement: Supplementary file 21 — Source data Fig. 3 [file 44318_2026_744_MOESM21_ESM.zip › Figure 3/B/FIGURE 3B/bandek_20250618_ND_islet34_CHRNA3_40x_4_dapi.tif]

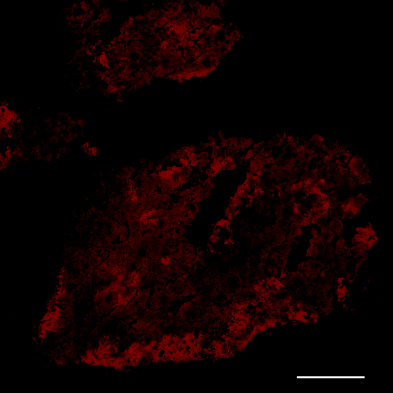

Supplement: Supplementary file 21 — Source data Fig. 3 [file 44318_2026_744_MOESM21_ESM.zip › Figure 3/B/FIGURE 3B/bandek_20250618_T2D_islet71_CHRNA3_40x_1_cpep.tif]

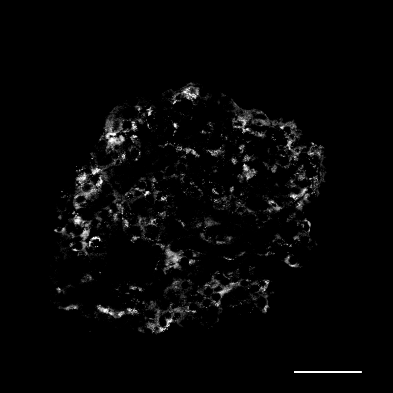

Supplement: Supplementary file 21 — Source data Fig. 3 [file 44318_2026_744_MOESM21_ESM.zip › Figure 3/B/FIGURE 3B/bandek_20250618_ND_islet34_CHRNA3_40x_4_gcg.tif]

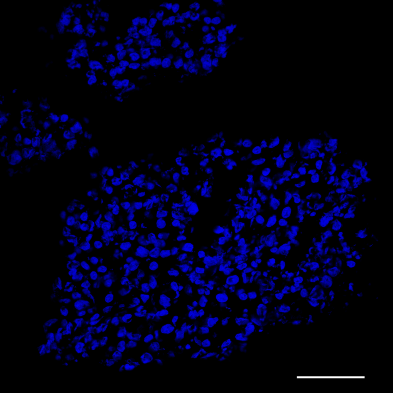

Supplement: Supplementary file 21 — Source data Fig. 3 [file 44318_2026_744_MOESM21_ESM.zip › Figure 3/B/FIGURE 3B/bandek_20250618_T2D_islet71_CHRNA3_40x_1_dapi.tif]

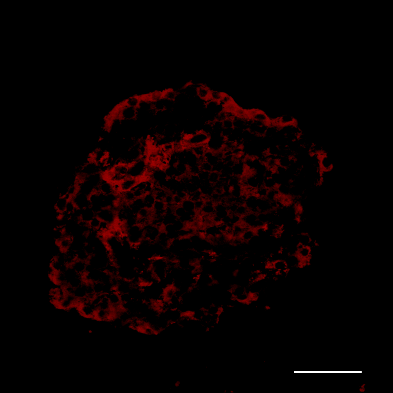

Supplement: Supplementary file 21 — Source data Fig. 3 [file 44318_2026_744_MOESM21_ESM.zip › Figure 3/B/FIGURE 3B/bandek_20250618_ND_islet34_CHRNA3_40x_4_cpep.tif]

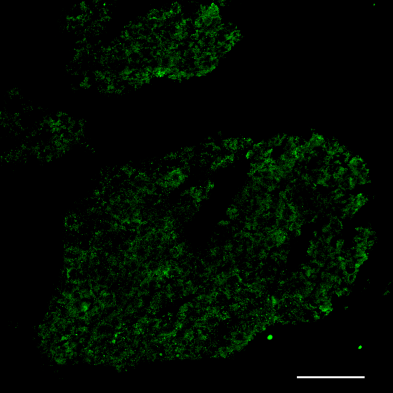

Supplement: Supplementary file 21 — Source data Fig. 3 [file 44318_2026_744_MOESM21_ESM.zip › Figure 3/B/FIGURE 3B/bandek_20250618_T2D_islet71_CHRNA3_40x_1_chrna3.tif]

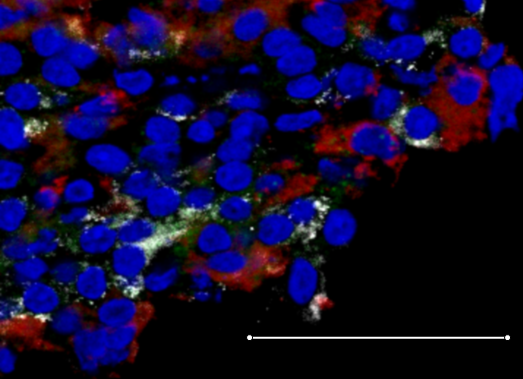

Supplement: Supplementary file 21 — Source data Fig. 3 [file 44318_2026_744_MOESM21_ESM.zip › Figure 3/B/FIGURE 3B/bandek_20250618_ND_islet34_CHRNA3_40x_4_zoom.tif]

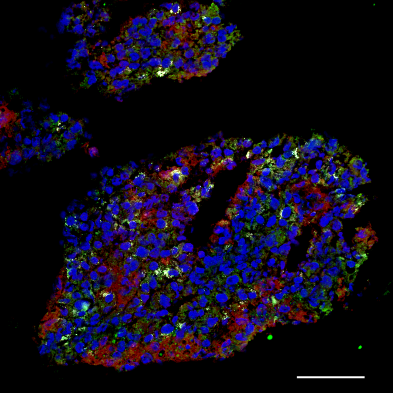

Supplement: Supplementary file 21 — Source data Fig. 3 [file 44318_2026_744_MOESM21_ESM.zip › Figure 3/B/FIGURE 3B/bandek_20250618_T2D_islet71_CHRNA3_40x_1_all.tif]

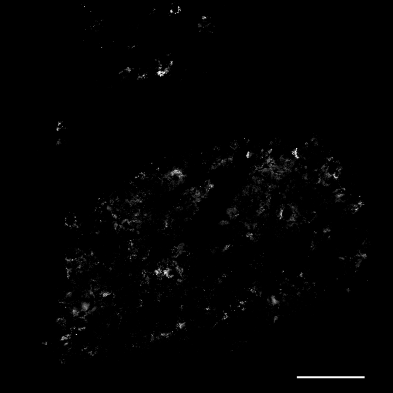

Supplement: Supplementary file 21 — Source data Fig. 3 [file 44318_2026_744_MOESM21_ESM.zip › Figure 3/B/FIGURE 3B/bandek_20250618_T2D_islet71_CHRNA3_40x_1_gcg.tif]

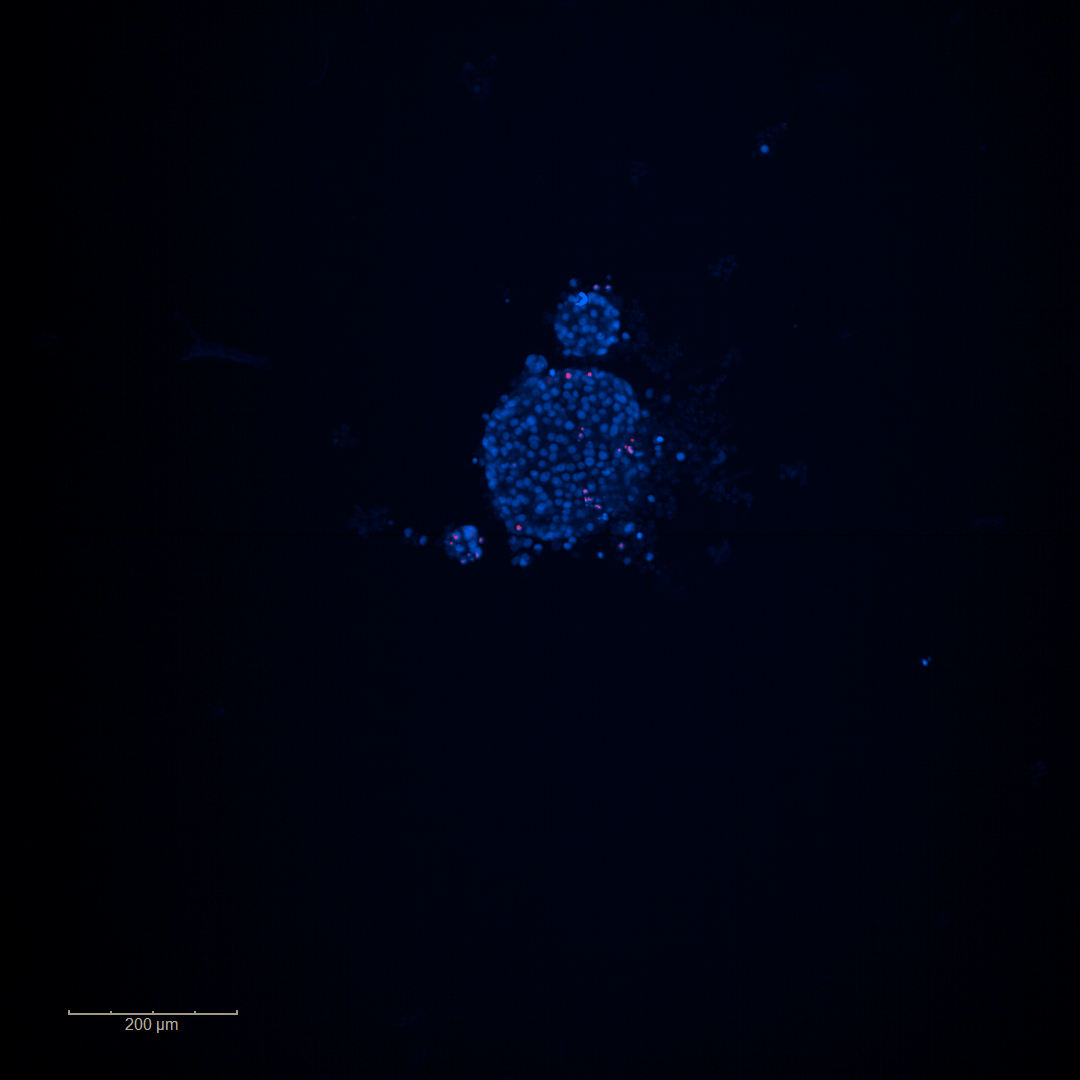

Supplement: Supplementary file 22 — Source data Fig. 4 [file 44318_2026_744_MOESM22_ESM.zip › Figure 4/F/islet227_PDZK1_F10_PI.tiff]

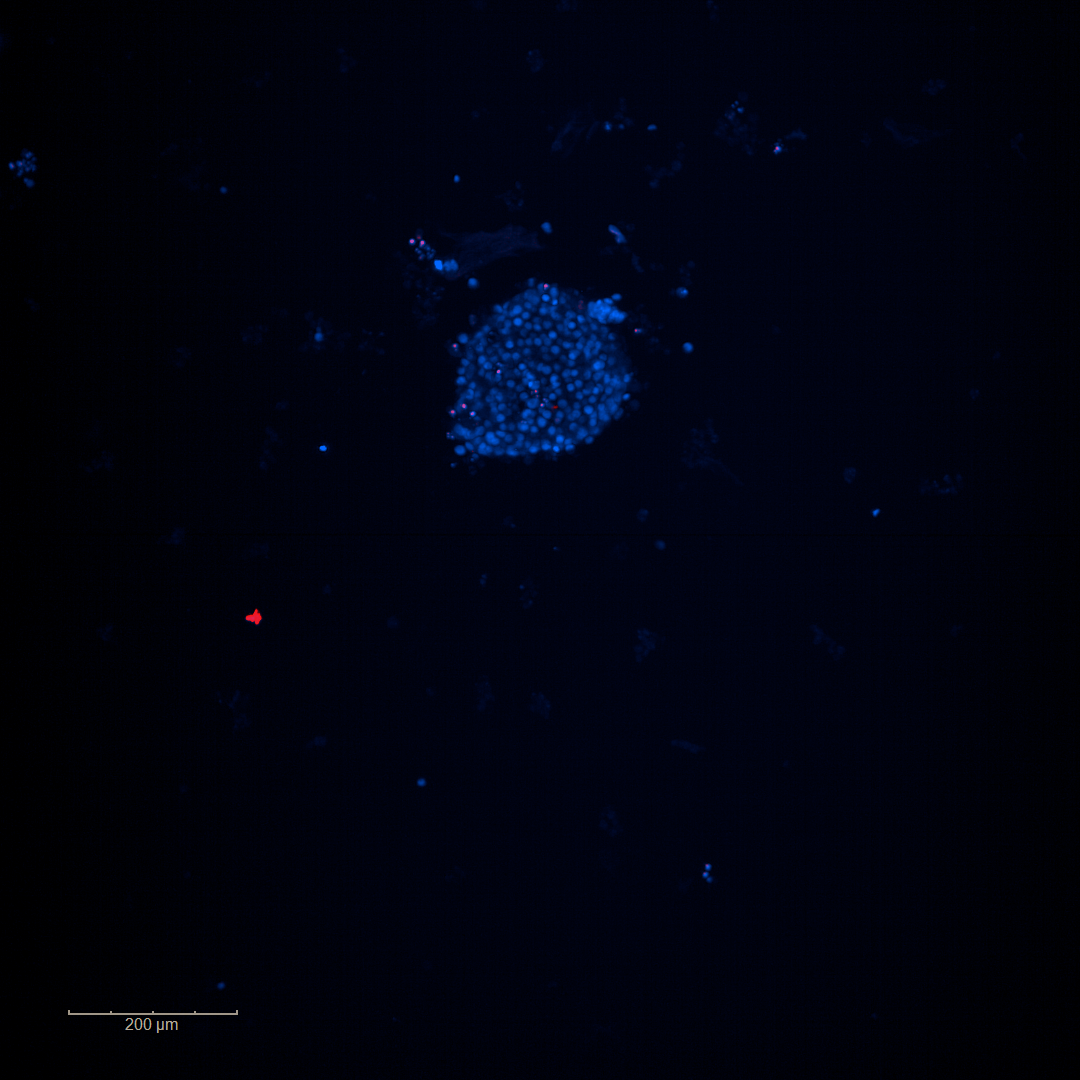

Supplement: Supplementary file 22 — Source data Fig. 4 [file 44318_2026_744_MOESM22_ESM.zip › Figure 4/F/Islet227_PITPNM2_B12_PI.tiff]

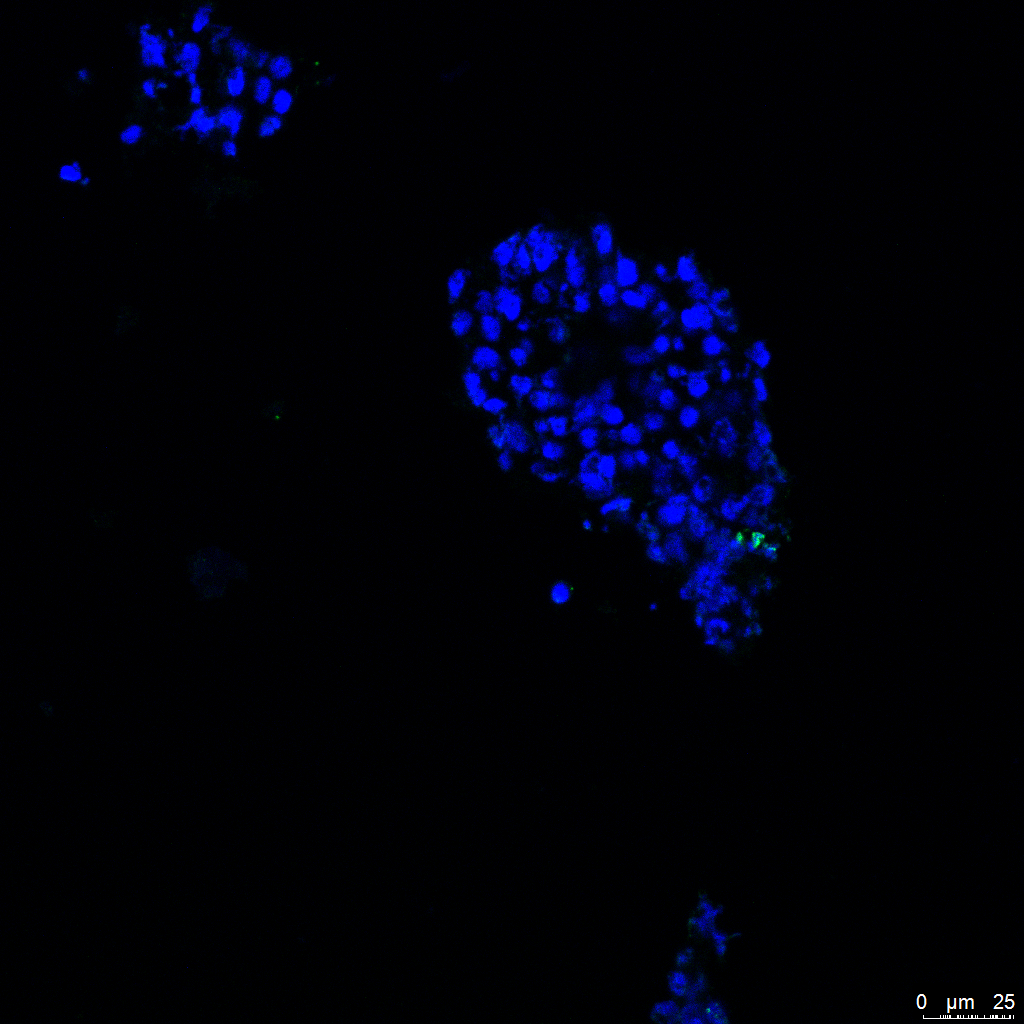

Supplement: Supplementary file 22 — Source data Fig. 4 [file 44318_2026_744_MOESM22_ESM.zip › Figure 4/H/Islet227_TUNEL_PITPNM2_4.tif]

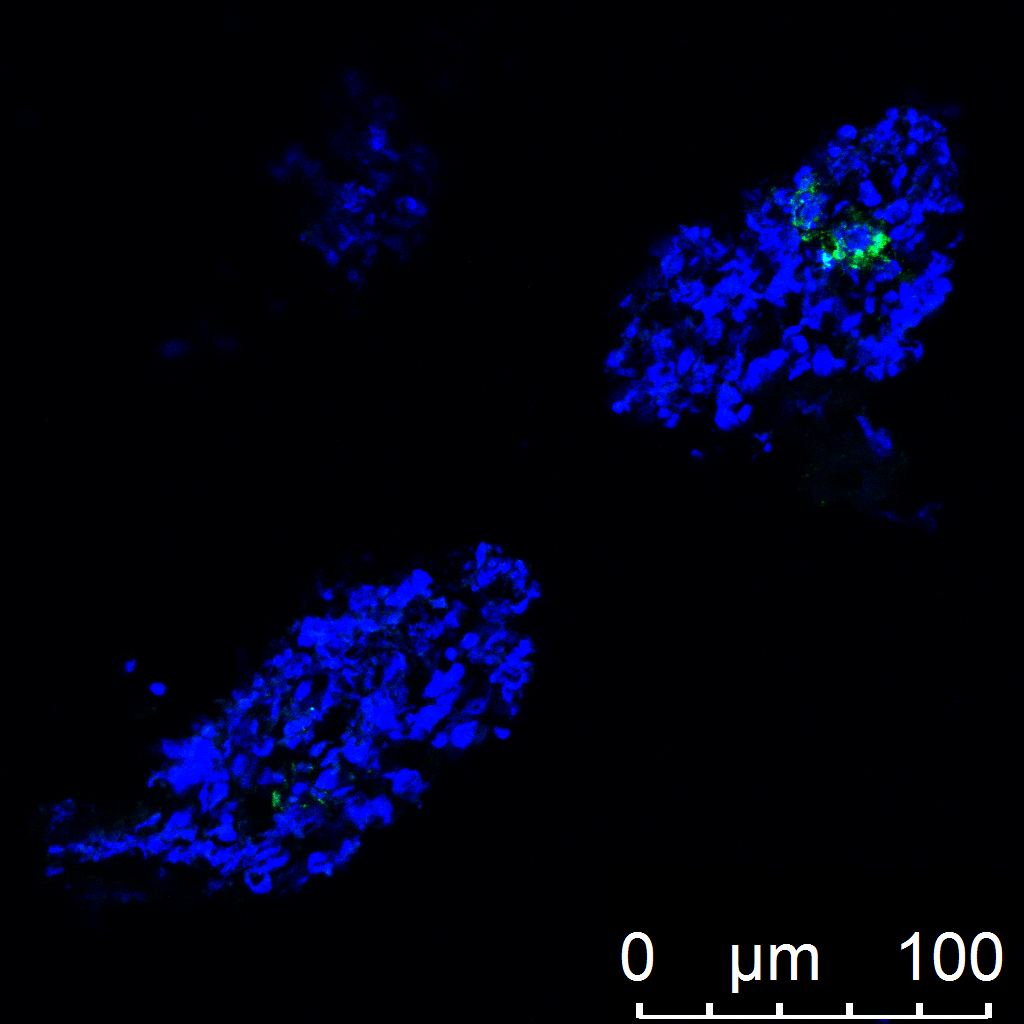

Supplement: Supplementary file 22 — Source data Fig. 4 [file 44318_2026_744_MOESM22_ESM.zip › Figure 4/H/Islet227_TUNEL_PDZK1_5.tif]

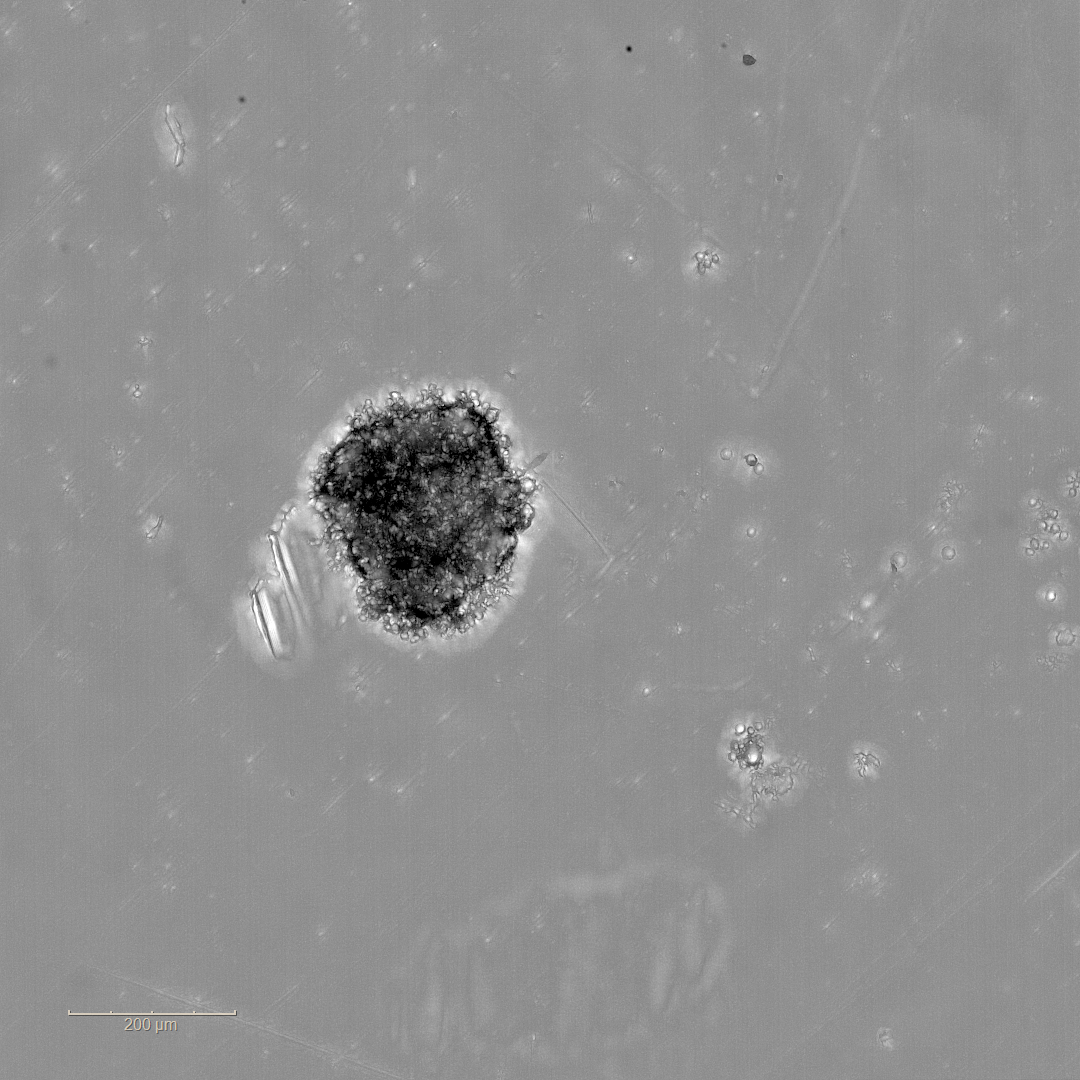

Supplement: Supplementary file 23 — Source data Fig. 5 [file 44318_2026_744_MOESM23_ESM.zip › Figure 5/I/Islet230_CTR_F3_max_proj.tiff]

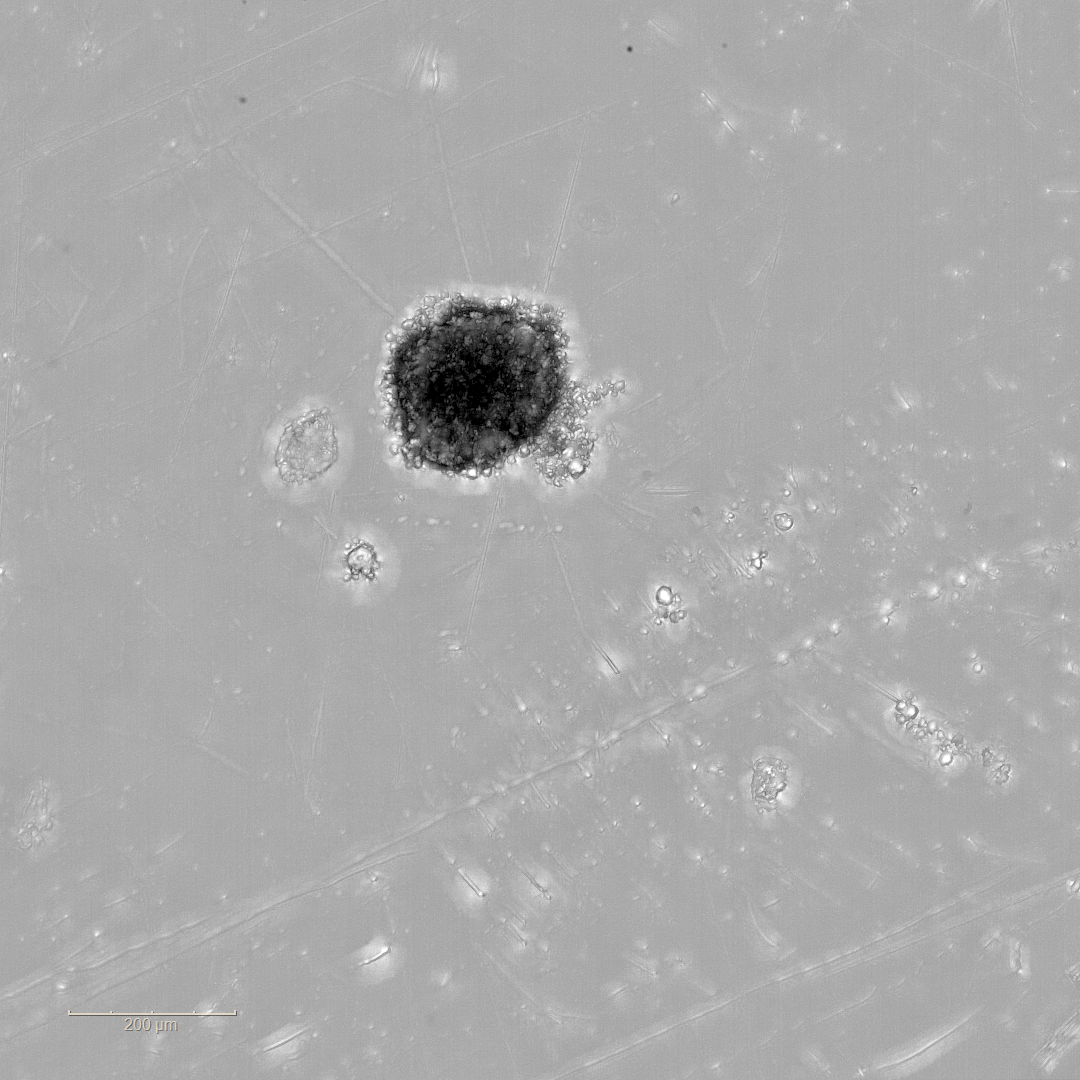

Supplement: Supplementary file 23 — Source data Fig. 5 [file 44318_2026_744_MOESM23_ESM.zip › Figure 5/I/Islet230_GRAMD3_G2_max_proj.tiff]
